# Population Pharmacokinetic Models of Voriconazole in Pediatric Patients: A Systematic Review

**DOI:** 10.3390/pharmaceutics18101341

**Published:** 2026-10-21

**Authors:** Xinyi Wang, Min Li, Xiyue Yuan, Jihui Chen

**Affiliations:** Department of Clinical Pharmacy, Xinhua Hospital, School of Medicine, Shanghai Jiao Tong University, Shanghai 200092, China; kath_wxy@sjtu.edu.cn (X.W.); crisoul@163.com (M.L.); yuanxiyue@xinhuamed.com.cn (X.Y.)

**Keywords:** voriconazole, population pharmacokinetics, pediatrics, therapeutic drug monitoring, model-informed precision dosing, systematic review

## Abstract

**Background/Objectives:** Voriconazole dosing in children is complicated by developmental and nonlinear pharmacokinetics, cytochrome P450 2C19 (CYP2C19) variation, variable oral bioavailability, inflammation, organ dysfunction, and drug interactions. We evaluated the characteristics, reporting completeness, and clinical applicability of pediatric and pediatric-inclusive voriconazole population pharmacokinetic (PopPK) models. **Methods:** PubMed, EMBASE, the Cochrane Library, Web of Science, and supplementary sources were searched from inception to 12 March 2026. Eligible studies developed a voriconazole PopPK model in patients aged 0–18 years or in a broader population with extractable or interpretable pediatric information. Reporting completeness was assessed using the 32-item Clinical Pharmacokinetic (ClinPK) checklist. **Results:** Eighteen studies published from 2004 to 2025 were included. Model-development cohorts contained 21–173 participants, and 15 studies reported 108–3336 concentrations. The median ClinPK reporting-completeness score was 82.6% (range, 74.2–90.0%). The included studies comprised one infant-specific, nine pediatric-only, six pediatric–young adult, and two mixed pediatric–adult models. Fifteen studies used parametric nonlinear mixed-effects methods and three used nonparametric methods; 15 selected two-compartment models and three selected one-compartment models. Body size was incorporated in 17 models and CYP2C19 phenotype was the most frequent retained covariate (10 models). Elimination structures, exposure targets, and dosing proposals were heterogeneous, and only one model underwent independent external validation. **Conclusions:** A population-matched model may support initial dosing, but early therapeutic drug monitoring and iterative Bayesian updating remain necessary. Current evidence does not support a universal pediatric model. Independent external validation and prospective implementation studies are required before routine clinical use.

## 1. Introduction

Invasive fungal diseases cause substantial morbidity and mortality in immunocompromised children, particularly those with hematological malignancies, hematopoietic cell transplantation (HCT), prolonged neutropenia, intensive chemotherapy, or critical illness [1,2,3]. Voriconazole, a second-generation triazole antifungal agent with broad activity against Aspergillus species and other clinically relevant molds, is a key agent for the treatment and prophylaxis of invasive fungal infections [2,4]. Both the Infectious Diseases Society of America (IDSA) and the European Society for Clinical Microbiology and Infectious Diseases–European Confederation of Medical Mycology (ESCMID-ECMM) guidelines recognize voriconazole as a key treatment option for pediatric invasive aspergillosis, although much of the efficacy evidence is derived from adults and data in neonates and infants remain limited [1,3,4]. Voriconazole dosing in children remains challenging because pediatric patients often exhibit higher weight-normalized clearance than adults, age-dependent metabolic capacity, variable gastrointestinal absorption, and marked interindividual pharmacokinetic variability [4,5]. These differences are reflected in current dosing recommendations. The IDSA guideline notes that children weighing <50 kg generally require higher voriconazole doses and that drug monitoring is particularly important in this population. It also highlights that oral bioavailability is lower and more variable in children than in adults, with reported estimates of approximately 50–65% in children compared with >95% in adults [4].

Therapeutic drug monitoring (TDM) is recommended for voriconazole because of its variable pharmacokinetics, clinically important drug interactions, and established exposure–response relationships [5,6]. However, trough-guided adjustment often begins after treatment initiation in routine practice and cannot fully anticipate developmental pharmacokinetics, capacity-limited metabolism, or CYP2C19-related differences [7,8]. Population pharmacokinetic (PopPK) modeling is a quantitative approach that is widely used to characterize pharmacokinetic variability and identify patient-specific determinants of drug exposure [9,10]. In pediatric pharmacology, PopPK modeling can analyze sparse and unbalanced concentration–time data while quantifying the effects of clinically relevant covariates, such as body weight, age, maturation, disease state, organ function, genotype, and concomitant medications [9,11]. When combined with Bayesian forecasting, PopPK models can further support model-informed precision dosing by integrating prior population information with individual TDM data to improve patient-specific exposure prediction [12,13]. However, although several PopPK models of voriconazole have been developed over the past two decades, it remains unclear whether any published model is sufficiently generalizable for routine use in pediatric populations.

The previous review included relatively few pediatric or pediatric-inclusive voriconazole models and predated several recent studies [8]. Accordingly, this review aimed to characterize these PopPK models, assess their reporting completeness and model-evaluation strategies, and evaluate how model structure, clinically important covariates, and dosing applications inform model selection and individualized voriconazole dosing in children.

## 2. Materials and Methods

This systematic review was conducted and reported in accordance with the Preferred Reporting Items for Systematic Reviews and Meta-Analyses 2020 (PRISMA 2020) statement [14]. The review was not registered, and no formal review protocol was prepared.

### 2.1. Literature Search Strategy

A systematic literature search was conducted to identify published studies reporting pediatric or pediatric-inclusive PopPK models of voriconazole. PubMed, EMBASE, the Cochrane Library, and Web of Science were searched from database inception to 12 March 2026 using terms related to voriconazole, pediatric populations, and PopPK modeling. Reference lists of eligible articles and relevant reviews were screened manually. Supplementary searches were conducted in *Therapeutic Drug Monitoring* and the conference proceedings of the *American College of Clinical Pharmacy*. All database, supplementary, and manual searches were completed on 12 March 2026. Complete search strategies are provided in Table S1.

### 2.2. Study Selection

Duplicate records were removed using a combination of automated and manual deduplication. Two reviewers independently screened titles and abstracts and then assessed potentially eligible full texts. Screening was managed using EndNote [15] and Covidence [16]. Disagreements were resolved by discussion, with consultation of a third reviewer when necessary.

Studies were eligible if they met all of the following criteria: (1) Population: pediatric patients aged 0–18 years, or a broader pediatric-inclusive population for which pediatric data, pediatric-specific parameters, or covariate effects were extractable or interpretable; (2) Treatment: voriconazole was administered by any route and at least one voriconazole concentration was measured; (3) Outcomes: reporting a final PopPK model structure and at least one key pharmacokinetic parameter, including elimination rate constant (k_e_), elimination half-life (t_1/2_), clearance (CL), volume of distribution (V_d_), central volume of distribution (V_1_), peripheral volume of distribution (V_2_), intercompartmental clearance (Q), absorption rate constant (k_a_), bioavailability (F), lag time (t_lag_), maximum elimination rate (V_max_), Michaelis–Menten constant (K_m_), intrinsic clearance (CL_int_); and (4) Analysis: PopPK modeling using parametric (nonlinear mixed-effects) or nonparametric approaches. Studies were excluded if they (1) did not report a PopPK modeling analysis; (2) included adult-only populations (>18 years) without extractable or interpretable pediatric data; (3) used only noncompartmental, individual pharmacokinetic, or conventional pharmacokinetic analyses; (4) were conference abstracts without sufficient methodological details or final model characteristics; (5) were reviews, study protocols, editorials, commentaries, letters, case reports, in vitro or animal studies; or (6) were published in non-English languages.

### 2.3. Data Extraction

Two reviewers independently extracted data from each included study using a standardized Microsoft Excel form. Extracted data were cross-checked, and discrepancies were resolved by discussion. The extracted information included: (1) study characteristics, including first author, year of publication, country or region, study design, data source, the sample size used for PopPK model development and, where applicable, independent external validation; (2) population characteristics, including age, sex, body weight, CYP2C19 metabolizer phenotypes, underlying disease, and special clinical populations. CYP2C19 metabolizer phenotypes were harmonized according to Clinical Pharmacogenetics Implementation Consortium (CPIC) definitions. Extensive metabolizers (EMs) were recoded as normal metabolizers (NMs), and heterozygous extensive metabolizers (HEMs) as intermediate metabolizers (IMs). Rapid metabolizers (RMs) and ultrarapid metabolizers (UMs) were retained as distinct categories unless the source study reported them as a combined group [7]; (3) voriconazole treatment and sampling characteristics, including route of administration, dose, dosing frequency, sampling strategy, number of concentrations, and bioanalytical assay; and (4) PopPK model characteristics, including modeling approach and software, structural model, absorption and elimination model, PK parameter estimates and units, interindividual, inter-occasion, and residual variability, covariates tested and retained, model evaluation methods, and model application.

### 2.4. Reporting Completeness Assessment

Two reviewers independently assessed reporting completeness using the 32-item ClinPK checklist, which covers six reporting domains: title/abstract, background, methods, results, discussion and conclusion, and other information, including funding sources and conflicts of interest [17,18]. Each item was scored as 1 if explicitly reported and 0 if not reported. Items considered not applicable were excluded from the assessment for the study, and the denominator was adjusted accordingly. The compliance score for each study was calculated as the number of reported items divided by the total number of applicable items and was expressed as a percentage. Disagreements were resolved by consensus. Because the ClinPK checklist focuses on reporting completeness, ClinPK scores were not interpreted as measures of model validity, clinical utility, or risk of bias. No formal study-level risk-of-bias assessment was undertaken because conventional risk-of-bias tools for intervention studies were not considered appropriate for this descriptive review of PopPK model-development studies, and we did not identify an accepted, validated PopPK-specific risk-of-bias instrument applicable to this setting. We therefore did not apply an intervention-study risk-of-bias tool or construct an unvalidated composite score. The detailed item-level ClinPK results are provided in Table S2. To complement the ClinPK assessment, methodological features relevant to model credibility, including development sample size, sampling richness, reporting of missing or excluded observations, parameter constraints, variability and residual error characterization, and internal and external model evaluation, were appraised descriptively.

### 2.5. Summary Measures

To summarize the study characteristics and PopPK analyses, continuous variables were described using means and standard deviations (SDs), medians and ranges, or values reported in the original studies, as appropriate. Categorical variables were summarized using frequencies and proportions. Because no meta-analysis or comparative effect synthesis was performed, formal assessments of reporting bias and certainty of evidence were not undertaken.

Each study contributed one final model to frequency summaries. Models were categorized according to the age composition of the modeling population as infant-specific models restricted to children aged <2 years, pediatric-only models in which all participants were aged ≤18 years, pediatric–young adult models with upper age limits >18 and ≤21 years, or mixed pediatric–adult models that included adults aged >21 years. Infant-specific and pediatric-only models were grouped as pediatric-restricted models, whereas pediatric–young adult and mixed pediatric–adult models were grouped as pediatric-inclusive models. When a study reported age-, genotype-, or formulation-specific parameter estimates, the study was counted once in model-frequency summaries, and subgroup-specific estimates were not treated as independent models. Model characteristics were summarized according to data source, route of administration, sampling design, modeling framework, disposition structure, absorption model, elimination parameterization, random-effects structure, covariate parameterization, model evaluation, and dosing application. All stratified comparisons were descriptive, and no formal between-group hypothesis testing was performed. Model evaluation methods were classified hierarchically as basic internal evaluation (goodness-of-fit and residual diagnostics), advanced internal evaluation (e.g., bootstrap, VPC/pcVPC, NPDE, SIR, cross-validation, or other predictive checks based on the model-development dataset), or internal evaluation plus external validation. External validation was defined as evaluation of the final PopPK model using an independent dataset that was not used for model development. Each study was assigned to the highest level of evaluation performed for the final PopPK model.

## 3. Results

### 3.1. Study Identification

**Figure 1:**
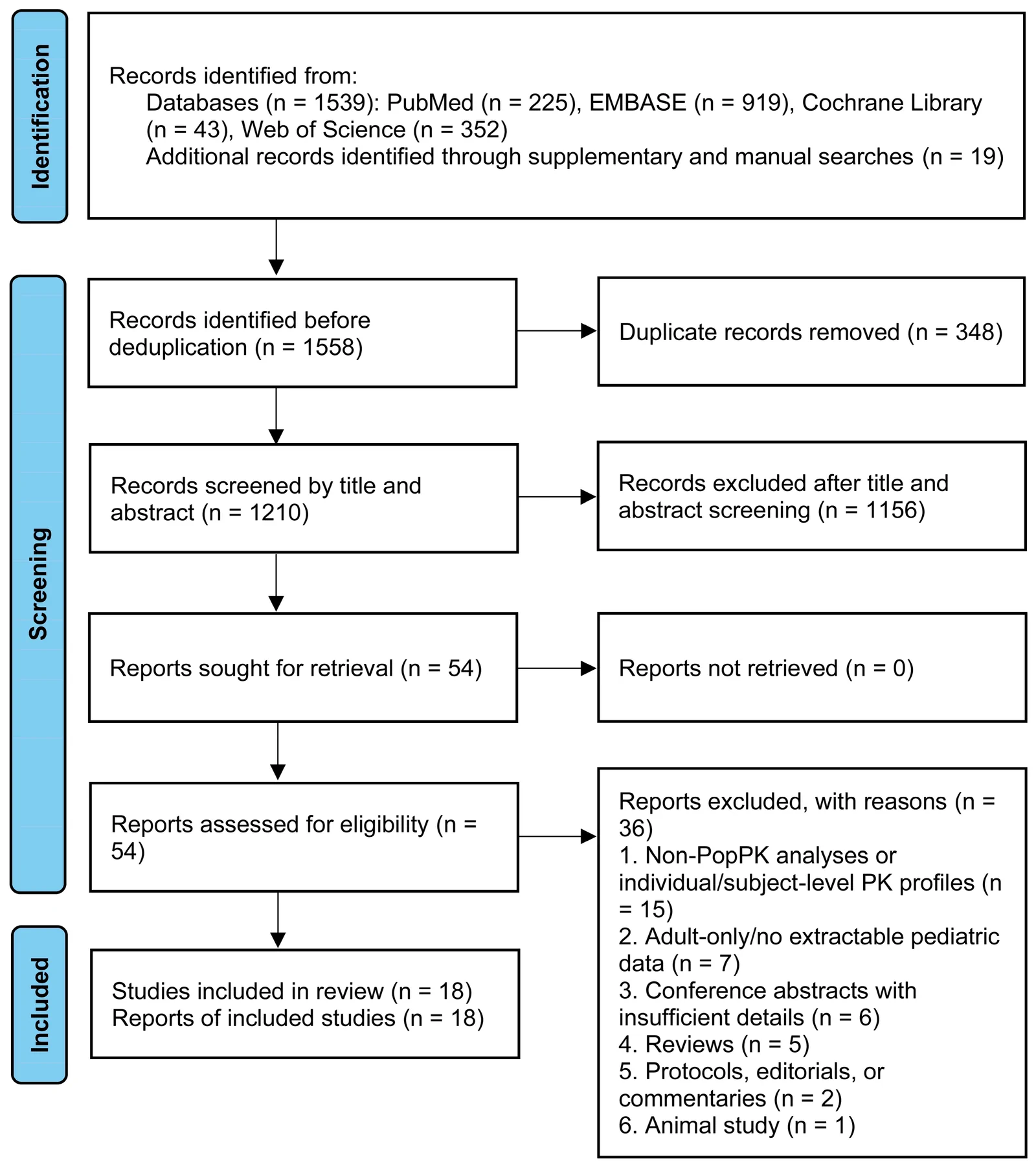
PRISMA 2020 flow diagram of the study selection process.

The study selection process is shown in Figure 1. Database searches identified 1539 records, including 225 from PubMed, 919 from EMBASE, 43 from the Cochrane Library, and 352 from Web of Science. An additional 19 records were identified through supplementary and manual searches. After removal of 348 duplicates, 1210 records underwent title and abstract screening. A total of 54 full-text articles were assessed for eligibility, of which 36 were excluded. The reasons for exclusion are summarized in Figure 1, and the individual reports excluded after full-text eligibility assessment and their primary reasons for exclusion are provided in Table S6. Finally, 18 studies [19,20,21,22,23,24,25,26,27,28,29,30,31,32,33,34,35,36] were included.

### 3.2. Reporting Completeness Assessment

The median ClinPK reporting-completeness score was 82.6% (range, 74.2–90.0%) (Table S2). Using 2015 as the cut-off year corresponding to the publication of ClinPK reporting recommendations, studies published in 2015 or later had a descriptively higher median compliance rate than earlier studies [83.9% (range 77.4–90.0%) vs. 78.1% (range 74.2–84.4%)]. The least frequently reported items were administration route in the title/abstract (5/18, 27.8%), sample storage (4/18, 22.2%), withdrawals or loss to follow-up when applicable (2/10, 20.0%), and the number of missing or excluded observations when applicable (8/17, 47.1%).

### 3.3. Study Characteristics

The 18 studies were published between 2004 and 2025. Model-development cohorts contained 21–173 participants (median, 59); total study cohorts contained up to 174 participants when an external-validation cohort was included. Fifteen studies reported 108–3336 voriconazole concentration measurements (median, 210). Eleven studies (61.1%) used prospectively collected data [20,24,25,26,27,29,30,31,33,35,36], whereas seven (38.9%) were retrospective observational studies [19,21,22,23,28,32,34]. The evidence comprised one (5.6%) infant-specific model [19], nine (50.0%) pediatric-only models [20,21,22,23,25,28,31,35,36], six (33.3%) pediatric–young adult models [24,26,27,29,32,34], and two (11.1%) mixed pediatric–adult models [30,33]. The most common clinical populations were immunocompromised or cancer patients in six studies (33.3%) [19,21,28,31,32,36], followed by patients undergoing HCT in four studies (22.2%) [20,24,26,27]. Voriconazole was administered by both intravenous and oral routes in 12 studies (66.7%) [20,21,22,23,28,29,30,31,32,33,34,35], intravenously only in five studies (27.8%) [24,25,26,27,36], and orally only in one study (5.6%) [19]. Blood sampling strategies varied substantially. Dense sampling, defined as more than five samples per patient, was used in seven studies (38.9%) [24,26,27,30,31,33,35], whereas trough-only sampling was used in six studies (33.3%) [19,21,22,23,32,34]. Three studies included both trough and peak concentrations [20,29,36], one used random concentrations [25], and one did not clearly specify the sampling strategy [28]. Bioanalytical methods for voriconazole quantification varied across studies. High-performance liquid chromatography (HPLC) was the most frequently used assay method (n = 7, 38.9%) [19,20,22,25,29,34,35], followed by liquid chromatography–tandem mass spectrometry (LC–MS/MS) (n = 6, 33.3%) [24,26,27,28,30,36], both methods in two studies (11.1%) [21,32], and enzyme-multiplied immunoassay technique (EMIT) in one study (5.6%) [23]. Summary characteristics are shown in Table 1, and individual study details are provided in Table S3.

### 3.4. Population Pharmacokinetic Analyses

The PopPK model characteristics are summarized in Table 1, Table 2 and Table S4. Nonlinear mixed-effects modeling (NLME) was the predominant approach, implemented in 15 studies (83.3%) [19,20,21,22,23,24,25,26,27,29,31,32,33,35,36], while the remaining three studies (16.7%) employed nonparametric adaptive grid (NPAG) algorithms [28,30,34]. Among the 15 parametric NLME models, the first-order conditional estimation with interaction (FOCE-I) was used in 10 studies [19,20,21,22,23,24,26,29,35,36], FOCE in two [31,33], and FOCE with extended least squares (FOCE-ELS) in one [25]; the estimation method was not clearly reported in the remaining two studies [27,32]. NONMEM was used in 12 studies (66.7%) [19,20,22,23,24,26,27,29,31,33,35,36], Phoenix NLME [21,25] and Pmetrics [28,30] in two each, and Monolix [32] and MM-USCPACK [34] in one each. All included studies reported at least one model evaluation procedure. According to the predefined hierarchical classification, 13 studies (72.2%) [19,20,21,22,23,24,25,26,29,31,33,35,36] performed advanced internal evaluation and four studies (22.2%) [27,28,32,34] reported only basic internal evaluation. Only one of the 18 PopPK models (5.6%) underwent independent external validation, using an independent cohort of 33 children [30]. Therefore, 17/18 models lacked evaluation in an independent dataset, and internal evaluation procedures were not considered evidence of model transportability.

Thirteen studies included oral data and described oral absorption using a first-order or delayed first-order process [19,20,21,22,23,28,29,30,31,32,33,34,35]. Across 13 studies, typical k_a_ values ranged from 0.43 to 1.53 h^−1^ in 6 studies [28,30,31,33,34,35], with 7 models fixing k_a_ at 0.849–1.20 h^−1^ [19,20,21,22,23,29,32]. A value of 1.19 h^−1^ was the most commonly used k_a_, appearing in 6 studies either as a fixed constant [19,20,22,23,29] or as an estimated parameter for the pediatric population [33]. Studies fixing k_a_ at 1.19 h^−1^ are based on estimates for the pediatric population reported by Friberg et al. [33]. Shoji et al. [21] fixed k_a_ at 1.20 h^−1^ after attempting estimation, also citing the work of Friberg et al. [33], whereas Hicks et al. [32] fixed k_a_ at 0.849 h^−1^, a value derived from the study by Karlsson et al. [35]. Friberg et al. was classified as an estimated-k_a_ study in the present pediatric/adolescent synthesis because k_a_ was estimated in children and adolescents (1.19 and 0.458 h^−1^, respectively). The rationale for fixing k_a_ varied across studies. Three studies explicitly related the use of a fixed k_a_ to trough-dominated sampling and limited information on the absorption phase [19,22,32]. Two studies cited limited sample size as a reason for fixing k_a_ [20,29]. Shoji et al. reported fixing k_a_ after attempting estimation, although the specific estimation-failure criterion was not reported [21]. Wu et al. (2022) used a literature-derived fixed value in an all-trough dataset but did not report a quantitative diagnostic of estimation failure [23]. Where an explicit rationale was unavailable, this was recorded as not reported rather than inferred. A lag time (t_lag_) was incorporated in 4 studies to characterize delayed absorption [21,31,33,34]. Three of these studies reported short delays of approximately 0.12 h [21,31,33]. Shoji et al. fixed t_lag_ at 0.12 h [21], Muto et al. estimated t_lag_ at 0.121 h using prior information [31], Friberg et al. estimated population-specific lag times, including approximately 0.120 h in children/adolescents [33]. One estimated substantially longer values, with t_lag_ estimates of 4.17 h for patients aged <12 years and 4.14 h for those ≥12 years [34]. Neely et al. removed the lag-time component during model development [30], Karlsson et al. assumed no lag time for oral administration [35], and the remaining seven studies did not provide sufficient information to characterize t_lag_. Twelve studies reported F estimates [20,21,22,23,28,29,30,31,32,33,34,35]. Among pediatric-restricted models, F ranged from 44.6% to 90.2% (median, 58.4%) [20,22,23,28,31,35], whereas pediatric-inclusive models reported values ranging from 44.6% to 85.0% [29,30,32,33,34]. Formulation differences were explicitly evaluated in 2/13 oral-data studies (15.4%). Friberg et al. considered powder for oral suspension versus tablet during model development and discussed formulation as a possible contributor to differences in k_a_ between age groups; however, formulation, age, and study differed concurrently and no independent formulation effect on k_a_ was retained [33]. Shoji et al. formally tested tablet versus dry syrup effects on k_a_, t_lag_, and F; formulation was retained only as a predictor of F [21].

Fifteen studies (83.3%) selected two-compartment models [20,21,24,25,26,27,28,29,30,31,32,33,34,35,36], whereas three studies (16.7%) [19,22,23] used one-compartment models. Two-compartment structures predominated among pediatric-only models and were used in all pediatric–inclusive models. The infant-specific model and two pediatric-only models used one-compartment models with linear elimination [19,22,23]. Among two-compartment models standardized to a 70 kg reference weight, the median (range) estimated values were 75.3 L (41.6–228 L) for V_1_, 101 L (48.9–1430 L) for V_2_, and 21.9 L/h (8.3–25.4 L/h) for Q [21,24,26,27,29,31,33]. Several pediatric-only and pediatric–young adult models used study-specific reference weights or direct weight-proportional equations, such as WT/20.5 kg [20], WT/18 kg [25], or direct WT-based expressions [28,32,34,35,36]. Elimination parameterization varied substantially across the included models. Linear elimination was used in six studies (33.3%) [19,20,22,23,24,36], nonlinear Michaelis–Menten elimination in seven (38.9%) [25,28,29,30,32,34,35], and mixed linear and nonlinear Michaelis–Menten in five (27.8%) [21,26,27,31,33]. These counts describe the selected model structures; they do not establish that saturable elimination is absent in the concentration range or population not represented by each dataset. Nonlinear elimination was generally described using Vmax and Km and the parameterization of metabolic capacity differed across studies. Of the 12 models with a nonlinear component, eight used time-invariant Vmax [25,26,27,28,29,30,32,34], three used a time-varying V_max_ or V_max,1_ [21,31,33], and one used an intrinsic-clearance formulation [35]. Across reported Michaelis–Menten parameterizations, Km ranged from 0.922 to 7.84 mg/L [25,28,29,30,32,34,35]. In the five mixed linear–nonlinear models standardized to a 70 kg reference weight, the median (range) CL was 6.02 L/h (4.60–7.70 L/h), while the median (range) V_max_ or V_max,1_ coefficient was 110 mg/h (36.2–118 mg/h) and the median (range) K_m_ was 1.20 mg/L (0.922–3.80 mg/L). For the distribution parameters in these mixed-elimination models, the median (range) V_1_, V_2_, and Q values were 75.3 L (52.4–79.0 L), 101 L (48.9–103.3 L), and 24.6 L/h (8.3–25.4 L/h), respectively [21,26,27,31,33]. These values represent model-specific reference coefficients rather than pooled pediatric estimates because three models incorporated time-dependent metabolic inhibition and the reference covariate conditions differed across studies [21,31,33].

Interindividual variability (IIV) was modeled using exponential random-effects in all 15 parametric models [19,20,21,22,23,24,25,26,27,29,31,32,33,35,36]; three additionally used additive random effects on the logit scale for F [29,31,33]. IIV was reported as CV%, variance, or SD and was therefore not uniformly comparable. Across models reporting IIV as CV%, the median IIV for CL was 61.8% (24.7–123.3%) [n = 10] [20,22,23,24,26,27,31,33,35,36]. The median (range) IIV for distribution parameters was 71.7% (7.74–169%) [n = 8] [24,25,26,27,29,31,32,33] for V_1_ and 77.0% (55.6–78.4%) [n = 3] [25,31,33] for V_2_. The median (range) IIV for Q was 55.2% (42.4–160.3%) [n = 4] [25,29,31,33]. In models with nonlinear or mixed elimination, the median (range) IIV for the maximum elimination rate (V_max_ or V_max,1_) was 38.7% (8.19–170%) [n = 8] [25,26,27,29,31,32,33], and the median (range) IIV for the K_m_ was 136% (131–136%) [n = 3] [31,33,35]. Variability in absorption-related parameters was less frequently reported. IIV in the k_a_ was reported in two studies, with values of 89.4% and 90.0% [31,33]. IIV for F was reported as 69.7% in one study [35] and was estimated using additive random effects on a logit scale in three studies [29,31,33]. Large IIV was reported for several parameters, and the metrics are not directly interchangeable across parameterizations. Inter-occasion variability (IOV) was reported in three studies [24,32,35]. IOV for CL was 41.2% and 43.0% in two studies [24,35], while one study [32] reported IOV values of 161% for V_1_ and 39% for V_max_.

A proportional residual error component was used to describe residual unexplained variability (RUV) in 13 models (72.2%) [20,21,22,23,24,25,26,27,29,30,31,33,35]. Among nine models reporting a single proportional RUV estimate explicitly on a percentage scale, the median (range) proportional residual error was 41.7% (10.0–94.7%) [20,22,23,25,26,27,29,30,31]. Shoji et al. reported a proportional residual error value of 0.7 without sufficient information to determine a directly comparable percentage scale [21], whereas Takahashi et al. reported an RUV estimate of 20.2 on an SD scale [24]; these values were therefore retained as reported but excluded from the percentage-based summary. Two models reported subgroup-specific proportional residual error values: Friberg et al. reported 37–59% in children and 36% in adolescents [33], whereas Karlsson et al. reported 57.3% in extensive metabolizers and 29.9% in heterozygous extensive or poor metabolizers [35]. One model additionally modeled IIV on RUV, with an estimated value of 142.6% [24]. Residual variability was unavailable or not directly comparable in five models, including the infant-specific model [19], two nonparametric models [28,34], and two older models [32,36]. No study used an additive residual error model alone.

Among 10 pediatric-restricted models, seven used two-compartment structures [20,21,25,28,31,35,36], whereas all eight pediatric-inclusive models used two-compartment structures [24,26,27,29,30,32,33,34]. Linear elimination was used in five pediatric-restricted models [19,20,22,23,36] but in only one pediatric-inclusive model. Nine pediatric-restricted models used parametric modeling approaches and described IIV using exponential random-effects models [19,20,21,22,23,25,31,35,36]. Among six pediatric-restricted models reporting IIV as CV% for clearance-related parameters, the median IIV for CL was 54.9% (range, 24.7–69.6%) [20,22,23,31,35,36]. A proportional residual error model was used in seven pediatric-restricted models [20,21,22,23,25,31,35]. Among five pediatric-restricted models reporting a single proportional residual error estimate explicitly on a percentage scale, the median was 47.6% (range, 23.9–94.7%) [20,22,23,25,31], whereas one model reported CYP2C19 phenotype-specific residual error values [35]. None of the pediatric-restricted PopPK models underwent independent external validation; the only externally validated model was developed using a mixed pediatric–adult population [30].

### 3.5. Body-Size Scaling and Covariate Effects

**Figure 2:**
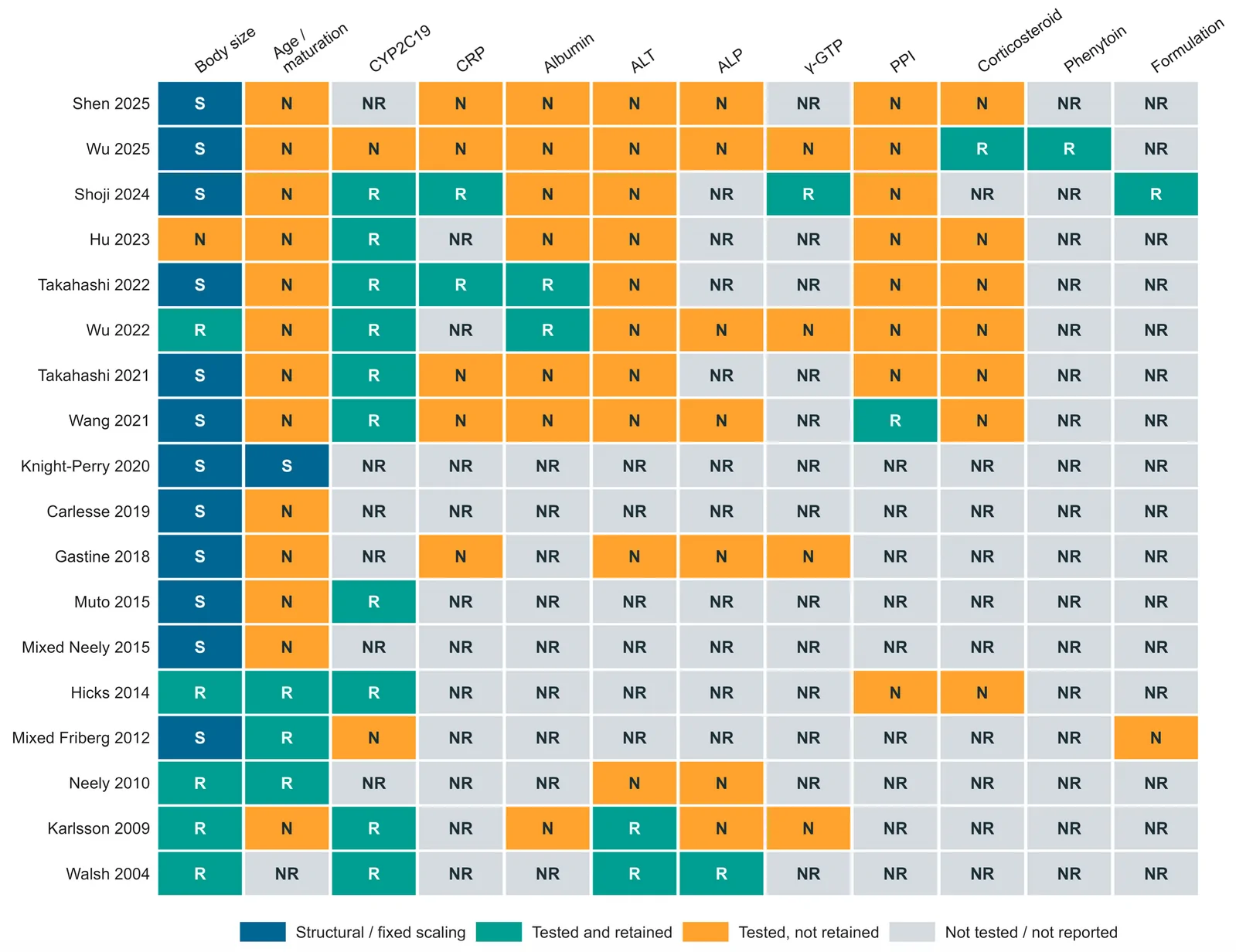
Covariate evaluation and retention across 18 studies [19,20,21,22,23,24,25,26,27,28,29,30,31,32,33,34,35,36]. S, structural or fixed scaling (body size; maturation where prespecified); R, tested and retained; N, tested but not retained; NR, not tested or not reported. For body size, 12 models used standard or near-standard allometric scaling, 5 retained other weight functions, and 1 tested but did not retain a weight effect. NR does not imply a null association. Abbreviations: ALB, albumin; ALP, alkaline phosphatase; ALT, alanine aminotransferase; CRP, C-reactive protein; CYP2C19, cytochrome P450 2C19; γ-GTP, γ-glutamyl transpeptidase; PPI, proton pump inhibitor.

Figure 2 summarizes the evaluation and retention status of major covariates across the 18 included models, distinguishing prespecified structural or fixed scaling, covariates tested and retained, covariates tested but not retained, and variables that were not tested or not reported.

Body size was incorporated into 17 models (94.4%) [19,20,21,23,24,25,26,27,28,29,30,31,32,33,34,35,36], either through prespecified allometric or proportional scaling or as an effect retained during formal covariate analysis. Twelve applied standard or near-standard allometric scaling to CL, Q, and/or V_max_, generally together with linear scaling of distribution volumes [19,20,21,24,25,26,27,28,29,30,31,33]. Direct weight-proportional or study-specific weight functions were used in five additional models [23,32,34,35,36]. Body weight scaled linear clearance or apparent oral clearance in 12 models [19,20,21,23,24,26,27,31,33,34,35,36]. In models incorporating Michaelis–Menten elimination, body weight also scaled V_max_ or V_max,1_ in 11 models [21,25,26,27,28,29,30,31,32,33,34].

CYP2C19 was explicitly evaluated in 12/18 studies (66.7%); it was retained in 10 and tested but not retained in two, while six studies did not explicitly report CYP2C19 evaluation and were therefore not interpreted as negative findings. As the most frequently retained non-size covariate, CYP2C19 metabolizer phenotype was retained in 10 models (55.6%) [21,22,23,24,25,26,31,32,35,36] and was linked exclusively to elimination-related parameters: linear or intrinsic clearance in six models [22,23,24,26,35,36] and V_max_ in five models [21,25,26,31,32]. It was retained in seven pediatric-restricted models [21,22,23,25,31,35,36] and three pediatric–young adult models [24,26,32]. Normal, rapid, or ultrarapid metabolizer phenotypes were generally associated with greater clearance or metabolic capacity, whereas intermediate and poor metabolizer phenotypes were associated with lower clearance, lower V_max_, or greater time-dependent inhibition of V_max_. Age was retained in four pediatric-inclusive models (4/18, 22.2%) but not in the pediatric-restricted models, in which developmental differences were generally represented indirectly through body-weight scaling [27,32,33,34]. These four pediatric-inclusive models included a maturation function on first-order clearance [27], an age-dependent exponent on V_max_, with increasing age reducing weight-normalized metabolic capacity [32], or age-stratified parameter sets (e.g., <12 vs. ≥12 years) covering absorption, bioavailability, distribution and elimination [33,34]. Biochemical covariates were retained less consistently and were incorporated into different structural parameters across models. Albumin was associated with linear clearance in two models, indicating lower model-predicted clearance at lower albumin concentrations [23,24]. Inflammation was implemented through different structural parameters: increasing CRP reduced linear clearance in one model [24], whereas CRP ≥2.0 mg/dL increased the time-dependent inhibition of V_max_ in another model [21]. An elevated γ-GTP grade also increased the time-dependent inhibition of V_max_ in the model developed by Shoji et al. [21]. ALT modified clearance or intrinsic clearance in two models [35,36], while ALP was additionally included as a modifier of linear clearance in one model [36]. Treatment-related covariates affected either elimination or oral bioavailability: phenytoin and methylprednisolone increased linear clearance in children undergoing allogeneic hematopoietic stem cell transplantation [20]; omeprazole reduced V_max_ in critically ill children [25]; dosage form modified oral bioavailability, with tablet administration associated with a lower F than dry syrup in the model developed by Shoji et al. [21].

### 3.6. Exposure Targets and Model-Informed Dosing Applications

Fifteen studies [19,20,21,22,23,25,26,27,29,31,32,33,34,35,36] provided model-informed dosing recommendations (Table S5) and 13 reported trough-based exposure targets: 11 specified bounded target ranges (0.5–5.0, 1.0–5.5, 1.5–5.0, or 1.0–6.0 mg/L) [19,20,22,23,25,26,27,28,29,30,32], whereas two specified lower-threshold targets only (≥1 mg/L) [21,34].

Four earlier models proposed regimens intended to match adult exposure or prior pediatric experience without specifying a single trough target [31,33,35,36]. Model-derived dose selection most consistently accounted for body size, age, and CYP2C19 metabolizer phenotype, generally supporting higher weight-normalized doses in younger or lower-weight children and lower doses in intermediate or poor metabolizers than in normal, rapid, or ultrarapid metabolizers [21,22,23,25,26,32]. More recent simulations additionally incorporated formulation and inflammatory or hepatic markers [21], albumin concentration [23], omeprazole coadministration [25], and phenytoin or methylprednisolone use [20]. Six studies specifically evaluated strategies to improve early exposure through modified loading schedules or temporarily intensified dosing [20,25,27,29,31,33]. However, the magnitude of dose adjustment and the probability of target attainment varied substantially, and most proposed regimens were not prospectively evaluated as clinical dosing algorithms [20,21,22,25,26]. These regimens should therefore be interpreted as population-specific model-derived dosing proposals rather than universally applicable pediatric recommendations. To translate these descriptive findings into clinically useful model screening, candidate models were mapped to major pediatric scenarios and model-selection needs in Table 3, which is a screening aid based on population and treatment compatibility. Compatibility can identify a candidate for evaluation; it does not establish that the model is validated for clinical dosing in that setting.

## 4. Discussion

### 4.1. Main Findings and Evidence Boundaries

This review identified 18 pediatric and pediatric-inclusive voriconazole PopPK models. The evidence supports population-matched model selection rather than a universal model because the studies differed in population characteristics and sampling designs, and were not compared in common external datasets. Body size was incorporated in 17/18 models (94.4%), and CYP2C19 phenotype was retained in 10/18 (55.6%). Among models reporting clearance-related IIV as CV%, the median was 61.8% (range, 24.7–123.3%; n = 10), indicating substantial residual between-patient variability. Thirteen of 18 models (72.2%) underwent advanced internal evaluation without independent external validation, whereas only 1/18 (5.6%) underwent independent external validation. These findings support using a compatible model to inform initial dosing, followed by early TDM and Bayesian updating, while emphasizing that population similarity alone does not establish predictive suitability.

Reporting completeness was descriptively higher after publication of the ClinPK recommendations [17,18], but important omissions persisted for sample storage, participant withdrawals, missing or excluded observations, and data-handling details. Importantly, ClinPK assesses reporting transparency rather than parameter identifiability, model overfitting, risk of bias, external predictive validity, or clinical utility. High checklist compliance should not be interpreted as evidence that a model is suitable for Bayesian dosing. Development cohorts ranged from 21 to 173 participants (median, 59), 6/18 studies relied on trough-only sampling, and missing or excluded observations were reported in only 8/17 applicable studies. These features do not establish a formal risk-of-bias category, but limited sampling information and incomplete reporting may constrain parameter identifiability and reproducibility, particularly for absorption and nonlinear parameters. Sample-size adequacy cannot be judged using a universal numerical threshold because it depends on model complexity, sampling design, concentration range, and parameter constraints.

### 4.2. Model Structure and Data Support

Fifteen studies used parametric NLME methods and three used nonparametric adaptive-grid methods. Parametric models generally characterize between-patient variability through prespecified continuous distributions and support formal covariate modeling and parameter uncertainty estimation [10,37]. Nonparametric methods do not require a predefined population parameter distribution and can represent skewed or multimodal distributions, which may be relevant when metabolic phenotypes or heterogeneous disease states create distinct subpopulations [37].

Model structure appeared to reflect dataset characteristics. All dense-sampling and intravenous-only studies selected two-compartment models, whereas trough-only studies were divided between one- and two-compartment structures. Among 10 pediatric-restricted models, seven used two-compartment structures, but elimination was divided equally between linear and nonlinear or mixed approaches. By comparison, all eight pediatric-inclusive models used two-compartment structures and only one used linear elimination. This descriptive contrast shows that neither two-compartment disposition nor nonlinear elimination was determined exclusively by the inclusion of participants older than 18 years; however, it should not be interpreted as evidence that voriconazole elimination is intrinsically more linear in children, given substantial differences in sampling intensity, route, dose and concentration ranges, parameter constraints, and the contribution of older-participant data to parameter identification. Independent mechanistic evidence also indicates quantitative developmental differences in voriconazole metabolism. Pediatric liver microsomes showed an approximately threefold higher apparent Vmax but a similar Km compared with adult microsomes, suggesting greater metabolic capacity in children without necessarily implying a different mathematical model form [38]. Nonlinear models require sufficient coverage of the concentration–elimination relationship to identify V_max_ and K_m_ separately [10,31]. When data are concentrated in the low-concentration region, V_max_ and K_m_ may be poorly identified as separate parameters, and their estimates become conditional on fixed values or prior information. Fixing K_m_ or applying prior information can improve numerical stability, but estimates of V_max_ and its covariate effects then become conditional on those assumptions. Similarly, time-varying V_max_ functions in pediatric models should be interpreted as empirical descriptions of changing apparent metabolic capacity rather than direct evidence of auto-inhibition [21,31,33]. Recent pediatric findings linking CYP2C19 phenotype, CRP, and γ-GTP to V_max_ inhibition support inflammation and hepatic status as contributors, but disease evolution and changing co-medications remain competing explanations [21,39,40]. Elimination models should therefore be compared using parameter precision and correlation, numerical stability, concentration-stratified simulation diagnostics, and external a priori and Bayesian predictive performance across the intended dose and exposure ranges [41]. Linear elimination can be an acceptable empirical approximation within a studied concentration range when parameter precision and predictive diagnostics support it. Because voriconazole is known to show saturable metabolism, a linear model should not be extrapolated to higher doses, concentrations, or populations without evaluation over that intended range. The six linear models in this review do not overturn the mechanistic rationale for nonlinear elimination; sparse concentration coverage and model constraints may limit identification of Vmax and Km, so structural choice must be judged against each dataset and external predictive performance.

Although the 13 models containing oral data generally used first-order or delayed first-order absorption, this predominance should be interpreted as a parsimonious empirical representation rather than evidence that absorption is physiologically uniform across pediatric ages, formulations, and clinical conditions [10]. The frequent use of fixed k_a_ values, particularly in sparse or trough-dominated datasets, is understandable because observations obtained near the end of the dosing interval contain little information on absorption rate [10,42]. However, fixing k_a_ makes early concentration predictions and estimates of t_lag_ conditional on the assumed absorption profile and limits the ability to distinguish rapid, delayed, or formulation-dependent absorption [10,43]. The repeated appearance of k_a_ values around 1.19 h^−1^ should not be interpreted as independent evidence of similar absorption rates in children across different studies. This apparent numerical similarity largely reflects the reuse of previously established parameter values rather than results derived from independent estimations. Moreover, k_a_ and t_lag_ characterize the timing of absorption, whereas F determines the fraction of the oral dose reaching the systemic circulation. When only oral data are available, CL/F and V/F cannot distinguish altered systemic disposition from reduced bioavailability, and reported F estimates should therefore be compared cautiously across models with different route information and parameter constraints. Earlier pediatric evidence estimated oral bioavailability at approximately 65%, and physiologically based pharmacokinetic (PBPK) modeling has suggested that intestinal first-pass metabolism may contribute to lower pediatric exposure, although this mechanism remains incompletely verified [44,45]. The identification of dosage form as a predictor of F in a recent pediatric model updates the earlier review, in which no significant pediatric bioavailability covariate had been identified, and indicates that formulation-specific differences should not be assumed to be negligible [8,21]. Food has also been shown to reduce oral voriconazole exposure in controlled studies, supporting prospective documentation of feeding conditions, although the magnitude of this effect should not be directly extrapolated from healthy adults to ill children [46].

### 4.3. Covariates for Initial Model Selection

Body-size effects were incorporated through either prespecified structural scaling or data-driven covariate selection. Fixed allometric or proportional functions standardize pharmacokinetic parameters to body size and provide a biologically informed model structure, whereas a statistically retained weight effect indicates that the selected structural relationship did not fully account for the observed size-associated variation [11,47]. However, body size cannot substitute for developmental maturation. Age was retained in only four pediatric-inclusive models and in none of the pediatric-restricted models, which may be attributable to the restricted age ranges in pediatric cohorts, the under-representation of infants, the high collinearity between age and weight, and the lack of sufficient data to estimate a separate maturation function. Age-dependent changes in CYP2C19 and flavin-containing monooxygenase 3 expression and activity provide biological plausibility for developmental differences in voriconazole metabolism, but the available evidence indicates that enzyme ontogeny is nonlinear and substrate dependent, particularly during infancy [48,49,50]. Accordingly, future models spanning infancy to adolescence should report the reference weight and scaling exponents, distinguish fixed from estimated size effects, examine age–weight collinearity, and evaluate continuous maturation functions when the age distribution and sampling information are sufficient. Broad age categories or simultaneous flexible weight and age functions should be used cautiously unless they improve parameter precision and out-of-sample prediction [11].

CYP2C19 genotype-predicted metabolizer phenotype was the most frequently retained covariate after body-size scaling. Nevertheless, the magnitude and clinical interpretation of the phenotype effect depended on the parameter to which it was linked. Differences in CYP2C19 retention may also reflect the alleles tested, phenotype-assignment rules, ancestry and phenotype frequencies, the number of rare rapid, ultrarapid, or poor metabolizers, concentration range, and statistical power. Pharmacogenetic guidance supports using CYP2C19 phenotype to identify patients at increased risk of subtherapeutic or excessive exposure, but genotype-predicted phenotype represents baseline metabolic potential rather than functional enzyme activity throughout treatment [7]. Inflammation can downregulate CYP2C19 activity and weaken the correspondence between genotype and observed phenotype, while enzyme inhibitors and inducers may cause additional phenoconversion [39,40]. Recent pediatric evidence also indicates that repeated TDM combined with CYP2C19 information is more informative than genotype alone [51]. CYP2C19 should therefore be used to inform initial dosing and early risk stratification, followed by concentration-guided Bayesian updating and reassessment of inflammatory, hepatic, and co-medication status. A model should not be favored solely because it has a more complex structure or contains more retained covariates, particularly when those covariates are unavailable, inconsistently measured, weakly identifiable, population-specific, or have not demonstrated improvement in out-of-sample predictive performance. Accordingly, clinical usefulness depends on reliable measurement at the relevant time point, applicability of the estimated effect to the intended target population, adequate identifiability of the linked pharmacokinetic parameter, and demonstrated improvement in independent or prospective predictive performance.

### 4.4. Time-Varying Clinical Factors

Some covariates are more useful for reassessment during therapy than for baseline model selection. Inflammatory, hepatic, and protein-binding markers were retained in relatively few models. These variables were measured at different frequencies, varied during treatment, and were linked to different structural parameters. Prospective studies in adults have shown that inflammation is associated with reduced voriconazole metabolism, while more recent evidence demonstrates inflammation-related weakening of the CYP2C19 genotype–activity relationship [39,40]. Hypoalbuminemia may alter the unbound fraction of voriconazole, but associations between albumin and total-concentration pharmacokinetics may also reflect hepatic dysfunction, systemic inflammation, capillary leakage, or disease severity [52]. These markers should therefore be interpreted as potentially time-varying indicators of physiological state rather than fixed baseline covariates. Future studies should synchronize serial CRP, albumin, and hepatic biochemical measurements with pharmacokinetic sampling, clearly define how time-varying values are carried forward or interpolated, and compare contemporaneous, delayed, and inter-occasion effects. Collinearity among inflammatory markers, hepatic tests, albumin, and severity indicators should also be evaluated to prevent unstable or clinically uninterpretable covariate relationships.

Co-medication and formulation effects require similarly parameter-specific and time-dependent interpretation. Binary exposure indicators provide limited information on dose, treatment duration, onset and offset of induction or inhibition, adherence, and timing relative to pharmacokinetic sampling [8]. Proton-pump inhibitors should not automatically be pooled as one covariate, because a prospective multicenter study found different effects of omeprazole and pantoprazole on voriconazole concentrations [53]. Corticosteroid exposure is particularly difficult to interpret because it may represent both a pharmacological interaction and a marker of transplantation status, inflammation, or disease severity, creating potential confounding by indication [20]. Mechanism-based classification into enzyme induction, enzyme inhibition, altered gastrointestinal absorption, and formulation-dependent bioavailability may improve interpretation, but individual drugs should remain separate when their effects are sufficiently represented.

### 4.5. Unexplained Variability and Limits of Covariate-Based Prediction

Substantial unexplained variability remained after covariate modeling. Across models reporting conventional parametric metrics, IIV in clearance-related parameters remained considerable, while variability in distribution, nonlinear elimination, absorption, and residual error was also substantial or inconsistently reported. These estimates may reflect biological heterogeneity but may also absorb errors in dose and sampling histories, unrecorded nonadherence or vomiting, route and formulation changes, assay error, handling of below-quantification-limit observations, structural misspecification, and inadequately modeled temporal changes [54,55]. The median clearance-related IIV of 54.9% in pediatric-restricted models is descriptive and should not be compared directly with pediatric-inclusive models because parameterizations and random-effects frameworks differed. IOV was evaluated in only three studies [24,32,35], although within-patient changes in clearance, F, inflammation, hepatic function, and co-medication exposure are plausible. Ignoring genuine IOV can bias IIV and RUV estimates, produce spurious temporal effects, distort model-derived exposure distributions, and create an overly optimistic impression of the precision achievable through TDM [35,56,57]. Future analyses should distinguish IIV, IOV, and RUV, define clinically meaningful occasions, report eta- and epsilon-shrinkage, and assess whether repeated treatment periods support IOV on CL, F, V_max_, or other time-varying parameters [57,58]. Residual error should not be treated as assay error alone, because it also represents dosing and sampling uncertainty and remaining structural inadequacy. Additional covariates should not be introduced solely to reduce unexplained variability until dose records, sampling times, below-quantification-limit handling, random-effects assumptions, and structural and residual error models have been examined. For Bayesian dosing, large IIV means population covariates may leave substantial uncertainty in an individual’s clearance or exposure. A sparse or single trough may not resolve that uncertainty, particularly for parameters with high IIV or weak identifiability. Initial doses from an unvalidated model can therefore miss target exposure or increase toxicity risk; early TDM, careful dose-history verification, and repeated updating remain important.

### 4.6. Model Selection, External Validation, and Clinical Implementation

Clinical translation should begin by defining the intended dosing task rather than by assuming that each diagnostic category requires a separate PopPK model. Models intended to support initial dosing should be screened for compatibility with the target age and weight range, disease context, route of administration, oral formulation, and availability of baseline covariates. Models intended for Bayesian dose adjustment should also accommodate routinely available concentration and clinical data during treatment [41,59]. Accordingly, the evidence map in Table 3 should be interpreted as a model-screening framework rather than a ranking of preferred models. It identifies candidate models for specific clinical scenarios according to population similarity, administration route, formulation, CYP2C19 phenotype availability, time-varying covariates, dosing application, and validation status. A model should not be favored solely because it has a more complex structure or contains more covariates, particularly when those covariates are unavailable, inconsistently measured, or not relevant to the intended dosing task. For example, an HCT-derived model may be prioritized for an HCT recipient, whereas an infant-specific model, a critical-care model, or a model incorporating oral formulation may warrant earlier consideration in the corresponding clinical setting. Such compatibility identifies candidates for evaluation but does not by itself establish clinical suitability.

Candidate models should subsequently be evaluated using representative independent data before local implementation. In this review, only one mixed pediatric–adult nonparametric model was evaluated in an independent cohort of 33 children, and no infant-specific or pediatric-only model underwent independent external validation [30]. Internal procedures such as bootstrap, VPC/pcVPC, and NPDE remain important for assessing parameter stability and predictive behavior within the development setting, but they do not establish transportability across institutions, assays, sampling strategies, or clinical populations [43,60]. External evaluation should assess both a priori predictions and Bayesian forecasts after incorporation of one or more measured concentrations. Prespecified evaluation criteria should include prediction bias and imprecision, predictive-interval coverage, classification of subtherapeutic and supratherapeutic exposure, and agreement between model-recommended and clinically feasible doses [41]. Model selection should therefore be based on clinically acceptable predictive performance and compatibility with the intended use, rather than goodness-of-fit results from the original development dataset alone. This evidence gap is particularly important when selecting an initial dose for medically fragile children, including patients with cancer, HCT recipients, critically ill children, and infants. A population-matched but externally unvalidated model may still mispredict clearance, bioavailability, or exposure when the target population differs from the development cohort in age, disease activity, CYP2C19 distribution, inflammation, co-medications, administration route, formulation, assay characteristics, or sampling practice. Such prediction error may result in subtherapeutic or supratherapeutic exposure before the first TDM result becomes available, with potential implications for treatment adequacy and toxicity. Bayesian updating after one or more measured concentrations can reduce subsequent prediction error, but it does not eliminate uncertainty arising from an inaccurate or poorly transportable prior model. The magnitude of these clinical risks could not be quantified from the included studies because they did not directly compare clinical outcomes between externally validated and unvalidated dosing models. This evidence gap supports local a priori and Bayesian predictive evaluation before implementation, together with early TDM during initial clinical use.

Because exposure targets, simulation assumptions, formulations, and dose increments differed across studies, published dosing proposals should not be averaged or transferred directly between pediatric populations. A pragmatic strategy is population-matched initial dosing followed by early TDM and Bayesian updating [61,62]. Clinically meaningful changes in inflammation, albumin, hepatic function, interacting medications, administration route, or formulation should trigger reassessment [21,24]. Before implementation, candidate models should be correctly encoded in dosing software and prospectively evaluated for target attainment, toxicity, feasibility, and workflow integration [63]. The near-term goal is therefore not a universal pediatric model but externally qualified, population-matched model-informed precision dosing pathways.

### 4.7. Limitations

This review has several limitations. First, the included studies differed substantially in population, age range, disease state, route of administration, sampling design, assay method, model structure, parameterization, and exposure targets, which limited direct quantitative comparison of pharmacokinetic parameters. Numerical summaries should therefore be interpreted as descriptive comparisons rather than pooled pediatric estimates. Second, eight included studies developed pediatric-inclusive models. Although these studies were included only when pediatric data, pediatric-specific model parameters or covariate effects were extractable or interpretable, the contribution of older participants may still limit pediatric-specific inference. Third, some studies did not fully report assay procedures, sampling details, model evaluation methods, residual error structures, missing-data handling, or sample management, limiting reproducibility and appraisal of clinical applicability. In addition, the ClinPK checklist assesses reporting completeness rather than methodological risk of bias. Because we did not identify an accepted, validated PopPK-specific risk-of-bias instrument applicable to this type of model-development review, no formal study-level risk-of-bias classification was assigned. Fourth, covariate summaries relied on variables retained in the final model. Failure to retain a covariate may reflect lack of availability, limited statistical power, collinearity, or a true null association and should not be interpreted uniformly as evidence of no clinical relevance. Fifth, independent external validation was scarce. Only one of the 18 PopPK models was evaluated in an independent cohort, and no infant-specific or pediatric-only model underwent independent external validation. Consequently, the transportability and a priori dosing performance of most models in new pediatric settings remain uncertain, particularly in fragile populations such as children with cancer or HCT recipients. Sixth, the review was not prospectively registered and no formal protocol was prepared, which may have increased susceptibility to post hoc methodological decisions, although study screening and data extraction were performed independently by two reviewers using predefined eligibility criteria and a standardized extraction process. Finally, exclusion of non-English studies may also have introduced language bias.

## 5. Conclusions

Published pediatric and pediatric-inclusive voriconazole PopPK models support population-matched rather than universal model selection. Body-size scaling was the most consistently incorporated structural feature, while maturation, CYP2C19 phenotype, inflammation, hepatic function, formulation, and concomitant medications may provide additional patient-specific information. Initial model-based dosing should be followed by early TDM and iterative Bayesian updating. Given limited independent external validation and heterogeneous exposure targets and dosing proposals, candidate models should undergo local predictive evaluation and prospective implementation testing before routine clinical use.

## Figures and Tables

**Table 1:** Summary characteristics of included pediatric and pediatric-inclusive voriconazole PopPK studies (n = 18).

Characteristic	Summary
Included studies	18
Publication years	2004–2025
Model-development sample size	Median 59, range 21–173
Reported concentration measurements per study	Median 210, range 108–3336; reported in 15 studies
Independent external validation	1 study; 33 children
Data collection	Prospective 11 (61.1%); retrospective 7 (38.9%)
Region	Asia 7 (38.9%); North America 7 (38.9%); Europe 3 (16.7%); South America 1 (5.6%)
Age composition	Infant-specific 1 (5.6%); pediatric-only 9 (50.0%); pediatric–young adult 6 (33.3%); mixed pediatric–adult 2 (11.1%)
Primary clinical setting	Immunocompromised/cancer 6 (33.3%); HCT 4 (22.2%); hematological malignancy non-HCT 2 (11.1%); critically ill 1 (5.6%); mixed/other ^a^ 5 (27.8%)
Administration route	Oral only 1 (5.6%); intravenous only 5 (27.8%); both intravenous and oral 12 (66.7%)
Blood sampling strategy	Dense sampling (>5 samples per patient) 7 (38.9%); trough-only 6 (33.3%); trough-and-peak 3 (16.7%); random 1 (5.6%); unspecified 1 (5.6%)
Assay method	HPLC only 7 (38.9%); LC–MS/MS only 6 (33.3%); HPLC and LC–MS/MS 2 (11.1%); EMIT 1 (5.6%); unspecified 2 (11.1%)
Modeling framework	Parametric NLME 15 (83.3%); nonparametric adaptive grid 3 (16.7%)
Main software	NONMEM 12 (66.7%); Phoenix NLME 2 (11.1%); Pmetrics 2 (11.1%); Monolix 1 (5.6%); MM-USCPACK 1 (5.6%)
Disposition model	One-compartment 3 (16.7%); two-compartment 15 (83.3%)
Elimination model	Linear 6 (33.3%); nonlinear Michaelis–Menten 7 (38.9%); mixed linear and nonlinear 5 (27.8%)
Oral absorption component	13 studies included oral data; k_a_ was fixed in 7 and estimated in 6; F was reported in 12
Body-size covariate/scaling ^b^	17 (94.4%)
CYP2C19 metabolizer phenotype	10 (55.6%)
Other retained covariates	Age 4; ALB, CRP, and ALT 2 each; ALP, γ-GTP, dosage form, omeprazole, phenytoin, and methylprednisolone 1 each
Model evaluation ^c^	Basic internal only 4 (22.2%); advanced internal only 13 (72.2%); independent external validation 1 (5.6%)
Dosing/exposure application	15 studies provided dosing recommendations, dosing simulations, or exposure-target applications; 13 reported trough-based targets

Data are n (%) unless otherwise stated. a. Mixed/other includes healthy adults, mixed pediatric–adult populations without one predominant condition, and pediatric populations with unspecified disease. b. Body-size effects include allometric, proportional, and weight-normalized scaling. Study-level route, dose, and sampling details are provided in Table S3. c. Categories reflect the highest evaluation level; definitions are in Methods Section 2.5. Dense sampling means > 5 samples per participant. Abbreviations: ALB, albumin; ALP, alkaline phosphatase; ALT, alanine aminotransferase; CRP, C-reactive protein; CYP2C19, cytochrome P450 2C19; EMIT, enzyme-multiplied immunoassay technique; F, oral bioavailability; HCT, hematopoietic cell transplantation; HPLC, high-performance liquid chromatography; ka, absorption rate constant; LC–MS/MS, liquid chromatography–tandem mass spectrometry; MM-USCPACK, multiple-model dosage design using USC*PACK; NLME, nonlinear mixed-effects modeling; NONMEM, nonlinear mixed-effects modeling software; NPAG, nonparametric adaptive grid; NPDE, normalized prediction distribution errors; pcVPC, prediction-corrected visual predictive check; PopPK, population pharmacokinetic; SIR, sampling importance resampling; TDM, therapeutic drug monitoring; VPC, visual predictive check; γ-GTP, γ-glutamyl transpeptidase.

**Table 2:** Population pharmacokinetic model parameter estimates and model variability.

Study	Structural Model	Pharmacokinetic Parameters	Model Variability		
			IIV	IOV	RUV
Shen 2025 [19]	One-compartment model with first-order absorption and elimination.	$\begin{array}{c} k_{a}\left(h^{-1}\right)=1.19\;\left(\mathrm{fixed}\right)\;\mathrm{CL}/F\;(L/h)=17.9\times {\left(WT/70\right)}^{0.75}\;V/F\left(L/70kg\right)=788\;F\;not\;provided. \end{array}$	CL/F: 0.674 (variance)	NR	Modeled; estimate NR
Wu 2025 [20]	Two-compartment model with first-order absorption and elimination.	$\begin{array}{c} k_{a}\left(h^{-1}\right)=1.19\left(\mathrm{fixed}\right)\;\;F=0.734\;Q\;\left(L/h\right)=11.5\times {\left(WT/20.5\right)}^{0.75}\;\mathrm{CL}\;(L/h)=3.61\times {\left(WT/20.5\right)}^{0.75}\times e^{1.12\times \mathrm{PHT}}\times e^{0.567\times \mathrm{MP}}\;V_{1}\left(L\right)=31.9\times \left(WT/20.5\right)\;V_{2}\left(L\right)=64\times \left(WT/20.5\right)\;\mathrm{Where}\;\mathrm{PHT}\;=\;1\;\mathrm{if}\;\mathrm{phenytoin}\;\mathrm{sodium}\;\text{co-administered},\;\mathrm{else}\;0;\;\mathrm{MP}\;=\;1\;\mathrm{if}\;\mathrm{methylprednisolone}\;\text{co-administered},\;\mathrm{else}\;0. \end{array}$	CL: 51.8%	NR	Proportional: 47.6%
Shoji 2024 [21]	Two-compartment model with first-order absorption and mixed linear and time-dependent nonlinear Michaelis–Menten elimination.	$\begin{array}{c} k_{a}\left(h^{-1}\right)=1.20\left(\mathrm{fixed}\right)\;\;\mathrm{logit}\left(F\right)=1.2-1.0\times I_{\mathrm{tablet}}\;t_{\mathrm{lag}}\left(h\right)=0.12\;\left(\mathrm{fixed}\right)\;Q\left(L/h\right)=24.6\times {\left(WT/70\right)}^{0.75}\;V_{max,1}\left(\mathrm{mg}/h\right)\;=\;110\times {\left(WT/70\right)}^{0.75}\;\mathrm{logit}\left(V_{max,\mathrm{in}h}\right)=2.0+0.5\times I_{\mathrm{IM}}+8.4\times I_{\mathrm{PM}}+0.8\times I_{\gamma \text{-}\mathrm{GTP}\geq 2}+2.7\times I_{\mathrm{CRP}\geq 2.0}\;T_{50}\left(h\right)=2.4\;\left(\mathrm{fixed}\right)\;V_{\max}\left(t\right)=V_{\max,1}\times \left(1-V_{\max,\mathrm{in}h}\times \left(1-e^{-\frac{\ln2}{T_{50}}\cdot t}\right)\right)\;K_{m}\left(\mathrm{mg}/L\right)=1.2\;CL\left(L/h\right)=7.7\times {\left(WT/70\right)}^{0.75}\;\;V_{1}\left(L\right)=75.3\times \left(WT/70\right)\;\;V_{2}\left(L\right)=103.3\times \left(WT/70\right)\;\;\mathrm{Where}\;I_{\mathrm{tablet}}=1\;\mathrm{if}\;\mathrm{tablet},\;\mathrm{else}\;0;\;\;I_{\mathrm{IM}}=1\;\mathrm{if}\;\mathrm{CYP}2C19\;\mathrm{intermediate}\;\mathrm{metabolizer},\;\mathrm{else}\;0;\;\;I_{\mathrm{PM}}=1\;\mathrm{if}\;\mathrm{CYP}2C19\;\mathrm{poor}\;\mathrm{metabolizer},\;\mathrm{else}\;0;\;I_{\gamma \text{-}\mathrm{GTP}\geq 2}=1\;\mathrm{if}\;\gamma \text{-}\mathrm{GTP}\;\mathrm{grade}\geq 2,\;\mathrm{else}\;0;\;I_{\mathrm{CRP}\geq 2.0}=1\;\mathrm{if}\;\mathrm{CRP}\geq 2.0\;\mathrm{mg}/\mathrm{dL},\;\mathrm{else}\;0. \end{array}$	Modeled; estimate NR	NR	Proportional: 0.7
Hu 2023 [22]	One-compartment model with first-order absorption and elimination.	$\begin{array}{c} k_{a}\left(h^{-1}\right)=1.19\left(\mathrm{fixed}\right)\;\;F=0.522\;\mathrm{CL}\;(L/h)=7.35\times I_{CYP2C19}\;V\left(L\right)=376\;\;\mathrm{Where}\;I_{CYP2C19}=1\;\mathrm{for}\;\mathrm{NM};\;I_{CYP2C19}=0.582\;\mathrm{for}\;\mathrm{IM};\;I_{CYP2C19}=0.381\;\mathrm{for}\;\mathrm{PM}. \end{array}$	CL: 24.7%; V: 233.2%	NR	Proportional: 94.7%
Wu 2022 [23]	One-compartment model with first-order absorption and elimination.	$\begin{array}{c} k_{a}\left(h^{-1}\right)=1.19\left(\mathrm{fixed}\right)\;F=0.902\;\mathrm{CL}\;\left(L/h\right)=2.29\times {\left(WT/23.7\right)}^{0.552}\times {\left(ALB/34.2\right)}^{2.15}+2.14\times I_{\mathrm{EM}}+0.624\times I_{\mathrm{IM}}\;V\left(L\right)=76\times \left(WT/23.7\right)\;\mathrm{Where}\;I_{\mathrm{EM}}=1\;\mathrm{if}\;\mathrm{CYP}2C19\;\mathrm{extensive}\;\mathrm{metabolizer},\;\mathrm{else}\;0;\;I_{\mathrm{IM}}=1\;\mathrm{if}\;\mathrm{CYP}2C19\;\mathrm{intermediate}\;\mathrm{metabolizer},\;\mathrm{else}\;0. \end{array}$	CL: 57%	NR	Proportional: 41.7%
Takahashi 2022 [24]	Two-compartment model with linear elimination.	$\begin{array}{c} Q\;\left(L/h\right)=16\times {\left(WT/70\right)}^{0.75}\;CL\left(L/h\right)=12.3\times {\left(WT/70\right)}^{0.75}\times I_{CYP2C19}\times e^{\left[0.32\times \left(ALB-2.8\right)\right]}\;\times \left[1-0.0025\left(CRP-27\right)\right]\;\;V_{1}\left(L\right)=41.6\times \left(WT/70\right)\;\;V_{2}\left(L\right)=59.1\times \left(WT/70\right)\;\;\mathrm{Where}\;I_{CYP2C19}=1\;\mathrm{for}\;\mathrm{EM};\;\;I_{CYP2C19}=0.67\;\mathrm{for}\;\mathrm{IM}/\mathrm{PM};\;\;I_{CYP2C19}=1.41\;\mathrm{for}\;\mathrm{RM}/\mathrm{UM}. \end{array}$	CL: 39.4%; V_1_: 118.1%; IIV on RUV: 142.6%	CL: 41.2%	Proportional: 20.2 (SD)
Wang 2021 [25]	Two-compartment model with nonlinear Michaelis–Menten elimination.	$\begin{array}{c} Q\;\left(L/h\right)\;=\;13.71\times {\left(WT/18\right)}^{0.75}\;V_{max}\;\left(\mathrm{mg}/h\right)=\;18.13\times {\left(WT/18\right)}^{0.76}\times e^{I_{CYP2C19}}\times e^{I_{\mathrm{omeprazole}}}\;K_{m}\left(\mathrm{mg}/L\right)=1.15\left(\mathrm{fixed}\right)\;\;V_{1}\left(L\right)\;=\;22.79\times \left(WT/18\right)\;V_{2}\left(L\right)\;\;=\;61.28\times \left(WT/18\right)\;\mathrm{Where}\;I_{CYP2C19}=0\;\mathrm{for}\;\mathrm{EM};\;I_{CYP2C19}=-0.12\;\mathrm{for}\;\mathrm{IM};\;I_{CYP2C19}=-0.38\;\mathrm{for}\;\mathrm{PM}; \end{array}$ $\mathrm{Where}\;I_{\mathrm{omeprazole}}\;=\;-0.21\;\mathrm{if}\;\mathrm{omeprazole}\;\text{co-administered},\;\mathrm{else}\;0.$	Q: 160.3%; V_max_: 8.19%; V_1_: 7.74%; V_2_: 55.59%	NR	Proportional: 58.2%
Takahashi 2021 [26]	Two-compartment model with mixed linear and nonlinear Michaelis–Menten elimination.	$\begin{array}{c} Q\;\left(L/h\right)\;=\;8.3\times {\left(WT/70\right)}^{0.75}\;V_{max}\;\left(\mathrm{mg}/h\right)\;=\;57.3\times {\left(WT/70\right)}^{0.75}\times I_{V}\;K_{m}\left(\mathrm{mg}/L\right)=3.80\;\mathrm{CL}\;\left(L/h\right)\;=\;5.8\times {\left(WT/70\right)}^{0.75}\times I_{CL}\;V_{1}\left(L\right)=75.9\times \left(WT/70\right)\;\;V_{2}\left(L\right)=48.9\times \left(WT/70\right)\;\mathrm{Where}\;I_{V}=0.6\;\mathrm{for}\;\mathrm{CYP}2C19=\mathrm{PM}/\mathrm{IM};\;I_{V}=1\;\mathrm{for}\;\mathrm{CYP}2C19=\mathrm{NM};\;I_{V}=1.1\;\mathrm{for}\;\mathrm{CYP}2C19=\mathrm{RM}/\mathrm{UM}\;\mathrm{Where}\;I_{CL}=0.76\;\mathrm{for}\;\mathrm{CYP}2C19=\mathrm{PM}/\mathrm{IM};\;I_{CL}=1\;\mathrm{for}\;\mathrm{CYP}2C19=\mathrm{NM};\;I_{CL}=1.6\;\mathrm{for}\;\mathrm{CYP}2C19=\mathrm{RM}/\mathrm{UM}. \end{array}$	CL: 77.4%; V_1_: 90.7%; V_max_: 43.0%	NR	Proportional: 28.2%
Knight-Perry 2020 [27]	Two-compartment model with mixed linear and nonlinear Michaelis–Menten elimination.	$\begin{array}{c} Q\;\left(L/h\right)=13.3\times {\left(WT/70\right)}^{0.75}\;V_{max}\;\left(\mathrm{mg}/h\right)=\;36.2\times {\left(WT/70\right)}^{0.75}\;K_{m}\left(\mathrm{mg}/L\right)=1.57\left(\mathrm{fixed}\right)\;CL\;\left(L/h\right)=4.60\times {\left(WT/70\right)}^{0.75}\times \frac{Age^{\gamma }}{Age^{\gamma }+1.21^{\gamma }}\;\;V_{1}\left(L\right)=52.4\times \left(WT/70\right)\;\;V_{2}\left(L\right)=86.7\times \left(WT/70\right)\;\;\mathrm{Where}\;\gamma \;\mathrm{is}\;\mathrm{the}\;\mathrm{Hill}\;\mathrm{coefficient}\;(\mathrm{assumed}\;\mathrm{to}\;\mathrm{be}\;1). \end{array}$	CL: 123.3%; V_1_: 111.1%; V_max_: 35.4%	NR	Proportional: 53.3%
Carlesse 2019 [28]	Two-compartment model with first-order absorption and nonlinear Michaelis–Menten elimination.	$\begin{array}{c} k_{a}\left(h^{-1}\right)=0.79\;F=0.48\;V_{max}\;\left(\mathrm{mg}/h\right)\;=\;1.24\times WT^{0.75}\;K_{m}\left(\mathrm{mg}/L\right)=5.36\left(\mathrm{fixed}\right)\;K_{cp}\;\left(h^{-1}\right)=0.49\;K_{pc}\;\left(h^{-1}\right)=0.09\;V_{1}\left(L\right)\;=0.67\times WT. \end{array}$	NA (nonparametric population distribution)	NR	NR
Gastine 2018 [29]	Two-compartment model with first-order absorption and nonlinear Michaelis–Menten elimination.	$\begin{array}{c} k_{a}\left(h^{-1}\right)=1.19\left(\mathrm{fixed}\right)\;F=0.594\;Q\;\left(L/h\right)\;=\;21.9\times {\left(WT/70\right)}^{0.75}\;V_{max}\;\left(\mathrm{mg}/h\right)\;=\;51.5\times {\left(WT/70\right)}^{0.75}\;K_{m}\left(\mathrm{mg}/L\right)=1.15\left(\mathrm{fixed}\right)\;\;V_{1}\left(L\right)=\;228\times \left(WT/70\right)\;V_{2}\left(L\right)=\;1430\times \left(WT/70\right). \end{array}$	F: 1.34 (logit-scale variance); V_1_: 45.4%; Q: 67%; V_max_: 63.6%	NR	Proportional: 37.8%; Additive: 0.0049 mg/L
Neely 2015 [30]	Two-compartment model with first-order absorption and nonlinear Michaelis–Menten elimination.	$\begin{array}{c} k_{a}\left(h^{-1}\right)=1.53\;\;F=0.85\;V_{max}\;\left(\mathrm{mg}/h\right)\;=\;1.82\times WT^{0.75}\;K_{m}\left(\mathrm{mg}/L\right)=1.54\;k_{cp}\;\left(h^{-1}\right)=0.40\;k_{pc}\;\left(h^{-1}\right)=0.15\;V_{1}\left(L\right)=1.20\times WT. \end{array}$	NA (nonparametric population distribution)	NR	Proportional: 10%; Additive: 0.29 mg/L
Muto 2015 [31]	Two-compartment model with first-order absorption and mixed linear and time-dependent nonlinear Michaelis–Menten elimination.	$\begin{array}{c} k_{a}\left(h^{-1}\right)=1.38\;\;t_{\mathrm{lag}}\left(h\right)=0.121\;\;F=0.645\;Q\left(L/h\right)=24.6\times {\left(WT/70\right)}^{0.75}\;\;V_{\max,1}\left(\mathrm{mg}/h\right)=118\times {\left(WT/70\right)}^{0.75}\;\;V_{\max,\mathrm{in}h}=0.93\;if\;CYP2C19\;is\;UM/EM;\;V_{\max,\mathrm{in}h}=1\;if\;CYP2C19\;is\;HEM/PM\;T_{50}\left(h\right)=2.45\;\;V_{\max}\;\left(\mathrm{mg}/h\right)=V_{\max,1}\times \left[1-\frac{V_{\max,\mathrm{in}h}\times \left(T-1\right)}{\left(T-1\right)+\left(T_{50}-1\right)}\right]\;K_{m}\left(\mathrm{mg}/L\right)=0.922\;CL\left(L/h\right)=6.02\times {\left(WT/70\right)}^{0.75}\;\;V_{1}\left(L\right)=75\times \left(WT/70\right)\;\;V_{2}\left(\;L\right)=101\times \left(WT/70\right). \end{array}$	k_a_: 89.4%; F: 1.69 (SD); V_1_: 14.2%; V_2_: 78.4%; Q: 43.4%; CL: 69.6%; V_max,1_: 170%; K_m_: 136%	NR	Proportional: 23.9%
Hicks 2014 [32]	Two-compartment model with first-order absorption and nonlinear Michaelis–Menten elimination.	$\begin{array}{c} k_{a}\left(h^{-1}\right)=0.849\left(\mathrm{fixed}\right)\;F=0.446\;Q\left(L/h\right)=0.609\times WT\;V_{max}\;\left(\mathrm{mg}/h\right)=\;3.36\times e^{-0.147\times Age\;+\;I_{CYP2C19}}\times WT\;K_{m}\left(\mathrm{mg}/L\right)=3\;V_{1}\left(L\right)\;=\;3.37\times WT\;V_{2}\left(\;L\right)\;=\;2.17\times WT\;\mathrm{Where}\;I_{CYP2C19}=1.273\;\mathrm{for}\;\mathrm{CYP}2C19*17/*17;\;I_{CYP2C19}=-0.147\;\mathrm{for}\;\mathrm{CYP}2C19*1/*17;\;I_{CYP2C19}=-0.444\;\mathrm{for}\;\mathrm{CYP}2C19*1/*2A\;\mathrm{or}\;*1/*2B;\;I_{CYP2C19}=-0.419\;\mathrm{for}\;\mathrm{CYP}2C19*2A/*2A;\;I_{CYP2C19}=0\;\mathrm{for}\;\mathrm{CYP}2C19*1/*1. \end{array}$	V_1_: 169%; V_max_: 42%	V_1_: 161%; V_max_: 39%	NR
Friberg 2012 [33]	Two-compartment model with first-order absorption and mixed linear and nonlinear (Michaelis–Menten and time-dependent V_max_) elimination.	$\begin{array}{c} k_{a}\;\left(h^{-1}\right)=1.19\;\left(\mathrm{child}\right)\;k_{a}\;\left(h^{-1}\right)=0.458\;\left(\mathrm{adolescent}\right)\;F=0.642\;t_{\mathrm{lag}}\;\left(h\right)=0.12\;Q\;\left(L/h\right)=25.4\times {\left(WT/70\right)}^{0.75}\;\;V_{\max,1}\;\left(\mathrm{mg}/h\right)=114\times {\left(WT/70\right)}^{0.75}\;V_{\max,\mathrm{in}h}=0.82\left(\mathrm{adolescent}\right)\;V_{\max,\mathrm{in}h}=0.75\;\left(\mathrm{child}\right)\;T_{50}\;\left(h\right)=2.41\;V_{\max}\;\left(\mathrm{mg}/h\right)=V_{\max,1}\times \left[1-\frac{V_{\max,\mathrm{in}h}\times \left(T-1\right)}{\left(T-1\right)+\left(T_{50}-1\right)}\right]\;K_{m}\;\left(\mathrm{mg}/L\right)=1.15\;CL\left(L/h\right)=6.16\times {\left(WT/70\right)}^{0.75}\;V_{1}\;\left(L\right)=79.0\times \left(WT/70\right)\;V_{2}\;\left(L\right)=103\times \left(WT/70\right). \end{array}$	k_a_: 90%; F: 1.67 (SD); V_1_: 14%; V2: 77%; Q: 42.4%; CL: 75%; V_max,1_: 24% (children),28% (adolescents); K_m_: 136%	NR	Children: 37–59%; adolescents: 36%; adults: 9% (IV), 16% (oral)
Neely 2010 [34]	Two-compartment model with absorption after a delay and Michaelis–Menten elimination.	$\begin{array}{c} k_{a}\;\left(h^{-1}\right)=0.51\;\left(<12\;\mathrm{years}\right),\;k_{a}\;\left(h^{-1}\right)=0.43\;\left(\geq 12\;\mathrm{years}\right)\;F=0.75\;\left(<12\;\mathrm{years}\right),\;F=0.81\;\left(\geq 12\;\mathrm{years}\right)\;t_{\mathrm{lag}}\;\left(h\right)=4.17\;\left(<12\;\mathrm{years}\right),\;t_{\mathrm{lag}}\;\left(h\right)=4.14\;\left(\geq 12\;\mathrm{years}\right)\;Q\;\left(L/h\right)=0.43\times WT\left(<12\;\mathrm{years}\right),\;Q\;\left(L/h\right)=0.68\times WT\left(\geq 12\;\mathrm{years}\right)\;V_{\max}\;\left(\mathrm{mg}/h\right)=0.9696\times WT\left(<12\;\mathrm{years}\right)\;V_{\max}\;\left(\mathrm{mg}/h\right)=1.568\times WT\left(\geq 12\;\mathrm{years}\right)\;K_{m}\;\left(\mathrm{mg}/L\right)=3.03\;\left(<12\;\mathrm{years}\right)\;K_{m}\;\left(\mathrm{mg}/L\right)=7.84\;\left(\geq 12\;\mathrm{years}\right)\;CL\;\left(L/h\right)=0.32\times WT\left(<12\;\mathrm{years}\right),\;CL\;\left(L/h\right)=0.20\times WT\left(\geq 12\;\mathrm{years}\right)\;V_{1}\;\left(L\right)=0.27\times WT\left(<12\;\mathrm{years}\right),\;V_{1}\;\left(L\right)=0.17\times WT\left(\geq 12\;\mathrm{years}\right)\;V_{2}\;\left(L\right)=2.34\times WT\left(<12\;\mathrm{years}\right),\;V_{2}\;\left(L\right)=0.83\times WT\left(\geq 12\;\mathrm{years}\right). \end{array}$	NA (nonparametric population distribution)	Not modeled	NR
Karlsson 2009 [35]	Two-compartment model with first-order absorption and Michaelis–Menten elimination.	$\begin{array}{c} k_{a}\left(h^{-1}\right)=0.849\;\;F=0.446\;Q\left(L/h\right)=0.609\times WT\;\;CL\;\left(L/h\right)=0.582\times WT\times \left(1-0.355\times I_{CYP2C19}\right)\times \left[1-0.093\times \left(\log\left(ALT\right)-\log\left(40.7\right)\right)\right]\;K_{m}\left(\;\mathrm{mg}/L\right)=3.03\;V_{1}\;\left(L\right)=\;0.807\times WT\;V_{2}\;\left(L\right)=\;2.17\times WT\;\mathrm{Where}\;I_{CYP2C19}=1\;\mathrm{for}\;\mathrm{HEM}/\mathrm{PM};\;I_{CYP2C19}=0\;\mathrm{for}\;\mathrm{EM}. \end{array}$	F: 69.7%; CL: 52.8%; K_m_: 131%	CL: 43%	Proportional: 57.3% (EM), 29.9% (HEM/PM)
Walsh 2004 [36]	Two-compartment model with linear elimination.	$\begin{array}{c} Q\left(L/h\right)=0.64\times WT\;\mathrm{CL}\;(L/h)=0.40\times \mathrm{WT}\times I_{CYP2C19}\times f\left(\mathrm{ALT}\right)\times f\left(\mathrm{ALP}\right)\;V_{1}\;\left(L\right)\;=\;0.8\times WT\;V_{2}\;\left(L\right)\;=\;\;1.7\;\times \;WT\;\mathrm{Where}\;I_{CYP2C19}=0.54\;\mathrm{for}\;\mathrm{HEM},\;I_{CYP2C19}=1\;\mathrm{for}\;\mathrm{EM};\;f\left(\mathrm{ALT}\right)\;and\;f\left(\mathrm{ALP}\right)\;not\;provided. \end{array}$	CL: 66.5%	NR	NR

Notes: Variability terms are presented separately as interindividual variability (IIV), inter-occasion variability (IOV), and residual unexplained variability (RUV). Numerical estimates are reported on the scale used in the original study and were not converted across CV%, variance, SD, or logit-scale parameterizations. Values reported on an unclear or non-percentage scale were retained as reported and were excluded from percentage-based cross-study summaries. “Modeled; estimate NR” means that the component was included but a numerical estimate was unavailable. “Not modeled” means that the component was explicitly absent from the reported final model. NR means that reporting was insufficient to determine the component or its estimate. NA means that a directly comparable parametric random-effects estimate is not applicable; for nonparametric models, NA in the IIV column does not imply absence of between-patient variability. Abbreviations: ALB, albumin; ALP, alkaline phosphatase; ALT, alanine aminotransferase; CL, clearance; CL/F, apparent oral clearance; CRP, C-reactive protein; CYP2C19, cytochrome P450 2C19; EM, CYP2C19 extensive metabolizer; F, oral bioavailability; γ-GTP, γ-glutamyl transpeptidase; HEM, CYP2C19 heterozygous extensive metabolizer; IIV, interindividual variability; IM, CYP2C19 intermediate metabolizer; IOV, inter-occasion variability; k_a_, absorption rate constant; k_cp_, first-order rate constant from the central to peripheral compartment; K_m_, Michaelis–Menten constant; k_pc_, first-order rate constant from the peripheral to central compartment; MP, methylprednisolone; NA, not applicable; NM, CYP2C19 normal metabolizer; PHT, phenytoin; PM, CYP2C19 poor metabolizer; Q, intercompartmental clearance; RM, CYP2C19 rapid metabolizer; RUV, residual unexplained variability; SD, standard deviation; T, time after the first dose; T50, time after treatment initiation at which half of the maximum inhibition occurred; tlag, lag time in drug absorption; UM, CYP2C19 ultrarapid metabolizer; V, volume of distribution; V/F, apparent oral volume of distribution; V_1_, central volume of distribution; V_2_, peripheral volume of distribution; V_max_, maximum elimination rate; V_max,1_, maximum elimination rate at 1 h after the start of dosing; V_max,inh_, maximum fractional inhibition of V_max_; WT, body weight. CV%, coefficient of variation expressed as a percentage.

**Table 3:** Clinical applicability evidence map of pediatric and pediatric-inclusive voriconazole PopPK models.

Clinical Scenario or Model-Selection Need	Candidate Models to Screen First	Main Boundary for Clinical Use and Limitations
Infants or children < 2 years	Shen 2025 [19]; broader models including very young children: Takahashi 2022 [24], Takahashi 2021 [26], Wang 2021 [25], Gastine 2018 [29]	Shen 2025 was the only infant-specific model. Broader models included young children within wider age ranges. No infant-specific model underwent independent external validation.
HCT	Wu 2025 [20]; Takahashi 2022 [24]; Takahashi 2021 [26]; Knight-Perry 2020 [27]	Most relevant to HCT settings, but they differ in route, age range, sampling design, covariates, and dosing purpose. None was externally validated.
Hematological malignancy, non-HCT	Hu 2023 [22]; Wu 2022 [23]	Relevant to hematological malignancy outside transplant-specific settings. Both used pediatric TDM-type data, but structural models and retained covariates differed.
Oncology or immunocompromised children	Shoji 2024 [21]; Carlesse 2019 [28]; Muto 2015 [31]; Hicks 2014 [32]; Walsh 2004 [36]	Relevant to oncology or immunocompromised populations, but disease context and treatment setting were heterogeneous. Route, formulation, CYP2C19 availability, and sampling design should be checked before use.
Critically ill children	Wang 2021 [25]	Only one model specifically targeted critically ill children. It incorporated CYP2C19 phenotype and omeprazole, but was based on intravenous data.
CYP2C19-informed initial model selection	Shoji 2024 [21]; Hu 2023 [22]; Takahashi 2022 [24]; Wu 2022 [23]; Takahashi 2021 [26]; Wang 2021 [25]; Muto 2015 [31]; Hicks 2014 [32]; Karlsson 2009 [35]; Walsh 2004 [36]	CYP2C19 was retained in 10 models but linked to different elimination-related parameters. Phenotype grouping, ancestry, rare phenotypes, route, inflammation, and co-medications differed across studies.
Oral therapy, bioavailability, or formulation considerations	Oral-component models; formulation effect retained in Shoji 2024 [21]	Oral data were included in 13 studies and F was reported in 12, but ka was often fixed. Only Shoji 2024 retained dosage form as a predictor of F.
Externally validated pediatric application	Neely 2015 [30]	Only model tested in an independent cohort of 33 children. However, it was a mixed pediatric–adult nonparametric model and does not replace validation of infant-, HCT-, oncology-, or ICU-focused models.

This table is intended as a model-screening aid and does not imply direct clinical transferability without local predictive evaluation. Abbreviations: CYP2C19, cytochrome P450 2C19; F, oral bioavailability; HCT, hematopoietic cell transplantation; ICU, intensive care unit; ka, absorption rate constant; PopPK, population pharmacokinetic; TDM, therapeutic drug monitoring.

## Data Availability

All data extracted and analyzed in this systematic review were derived from published studies and are provided in the article and its Supplementary Materials. No new datasets were generated.

## Supplementary Materials

The following supporting information can be downloaded at https://www.mdpi.com/article/10.3390/pharmaceutics18101341/s1, Table S1: Search strategies; Table S2: Reporting completeness assessment based on the ClinPK checklist; Table S3: Characteristics of individual studies; Table S4: Summary of population pharmacokinetic model development; Table S5: Summary of population pharmacokinetic model applications and dosing recommendations; Table S6: Full-text reports excluded after eligibility assessment and primary reasons for exclusion. The 18 studies summarized in Tables S2–S5 are cited in the main reference list [19,20,21,22,23,24,25,26,27,28,29,30,31,32,33,34,35,36]. PRISMA 2020 Checklist.
