# Supplementary material for: Population Pharmacokinetic Models of Voriconazole in Pediatric Patients: A Systematic Review
Source: Pharmaceutics. 2026 Oct 21;18(10):1341. doi: 10.3390/pharmaceutics18101341 (PMC13708257; doi:10.3390/pharmaceutics18101341)
Supplement: Supplementary file 1 [file pharmaceutics-18-01341-s001.zip › Supplementary Files/Supplementary_material.pdf]

Supplementary Materials

Population Pharmacokinetic Models of Voriconazole in Pediatric Patients: A Systematic Review

Xinyi Wang†, Min Li†, Xiyue Yuan, Jihui Chen*

Department of Clinical Pharmacy, Xinhua Hospital, School of Medicine, Shanghai Jiao Tong University, Shanghai 200092, China;

†These authors contributed equally to this work

*Correspondence:

Jihui Chen

Department of Clinical Pharmacy, Xinhua Hospital, School of Medicine, Shanghai Jiao Tong University, 1665 Kongjiang Road, Shanghai 200092, P.R.China. Email: chenjihui@xinhuamed.com.cn.

Table S1. Search strategies

**PubMed**	
1	("Voriconazole"[Mesh] OR voriconazole*[tiab] OR vfend*[tiab] OR vorikonazole*[tiab] OR vorikonazol*[tiab] OR voriconazol*[tiab])
2	(pediatric*[tiab] OR paediatric*[tiab] OR child*[tiab] OR stepchild*[tiab] OR newborn*[tiab] OR congenital*[tiab] OR infan*[tiab] OR baby[tiab] OR babies[tiab] OR neonat*[tiab] OR pre-term[tiab] OR preterm*[tiab] OR pre-matur*[tiab] OR prematur*[tiab] OR "birth weight"[tiab] OR "birth-weight"[tiab] OR birthweight*[tiab] OR "full term"[tiab] OR "full-term"[tiab] OR post-natal*[tiab] OR postnatal*[tiab] OR postterm[tiab] OR post-term[tiab] OR NICU[tiab] OR preschool*[tiab] OR pre-school*[tiab] OR junior*[tiab] OR toddler*[tiab] OR kid*[tiab] OR boy*[tiab] OR girl*[tiab] OR pubescen*[tiab] OR juvenile*[tiab] OR teen*[tiab] OR preteen*[tiab] OR pre-teen*[tiab] OR youth*[tiab] OR youngster*[tiab] OR adolesc*[tiab] OR school*[tiab] OR kindergarten*[tiab] OR pre-pubesc*[tiab] OR prepubesc*[tiab] OR puberty[tiab] OR minors[tiab] OR PICU[tiab]) OR "Adolescent"[Mesh:NoExp] OR "Child"[Mesh:NoExp] OR "Child, Preschool"[Mesh:NoExp] OR "Infant"[Mesh:NoExp] OR "Child, Hospitalized"[Mesh:NoExp]
3	("Pharmacokinetics"[Mesh] OR "Area Under Curve"[Mesh] OR "Tissue Distribution"[Mesh] OR "Models, Biological"[Mesh] OR "hepatobiliary elimination"[tiab] OR "renal elimination"[tiab] OR "patient-specific modeling"[tiab] OR "pharmacokinetic*"[tiab] OR "nonlinear mixed effect*"[tiab] OR "nonmem"[tiab] OR "WinNonlin"[tiab] OR "non-compartmental analys*"[tiab] OR "noncompartmental analys*"[tiab] OR "pharmacometric*"[tiab] OR "ADAPT 5"[tiab] OR "MONOLIX"[tiab] OR "population analy*"[tiab] OR "PDx-Pop"[tiab] OR "Pharsight"[tiab] OR "PK"[tiab] OR "PopKinetics"[tiab] OR "S-ADAPT"[tiab] OR "Simcyp"[tiab] OR "nlmixed"[tiab] OR "nlme"[tiab])
4	1 AND 2 AND 3
**EMBASE**	
1	'voriconazole'/exp OR voriconazol*:ti,ab,kw OR vorikonazol*:ti,ab,kw OR voriconazole:ti,ab,kw OR vfend*:ti,ab,kw
2	'adolescent'/exp OR 'child'/exp OR 'infant'/exp OR 'preschool child'/exp OR 'school child'/exp OR 'hospitalized child'/exp OR 'newborn'/exp OR 'pediatrics'/exp OR pediatric*:ti,ab,kw OR paediatric*:ti,ab,kw OR child*:ti,ab,kw OR stepchild*:ti,ab,kw OR newborn*:ti,ab,kw OR congenital*:ti,ab,kw OR infan*:ti,ab,kw OR baby:ti,ab,kw OR babies:ti,ab,kw OR neonat*:ti,ab,kw OR pre-term:ti,ab,kw OR preterm*:ti,ab,kw OR pre-matur*:ti,ab,kw OR prematur*:ti,ab,kw OR 'birth weight':ti,ab,kw OR 'birth-weight':ti,ab,kw OR birthweight*:ti,ab,kw OR 'full term':ti,ab,kw OR 'full-term':ti,ab,kw OR post-natal*:ti,ab,kw OR postnatal*:ti,ab,kw OR postterm:ti,ab,kw OR post-term:ti,ab,kw OR NICU:ti,ab,kw OR preschool*:ti,ab,kw OR pre-school*:ti,ab,kw OR junior*:ti,ab,kw OR toddler*:ti,ab,kw OR kid*:ti,ab,kw OR boy*:ti,ab,kw OR girl*:ti,ab,kw OR pubescen*:ti,ab,kw OR juvenile*:ti,ab,kw OR teen*:ti,ab,kw OR preteen*:ti,ab,kw OR pre-teen*:ti,ab,kw OR youth*:ti,ab,kw OR youngster*:ti,ab,kw OR adolesc*:ti,ab,kw OR school*:ti,ab,kw OR kindergarten*:ti,ab,kw OR pre-pubesc*:ti,ab,kw OR prepubesc*:ti,ab,kw OR puberty:ti,ab,kw OR minors:ti,ab,kw OR p?ediatric*:ti,ab,kw OR PICU:ti,ab,kw

3	'pharmacokinetics'/exp OR 'area under the curve'/exp OR 'tissue distribution'/exp OR 'biological model'/exp OR 'renal excretion'/exp OR 'bile excretion'/exp OR 'population pharmacokinetics'/exp OR 'nonlinear mixed effect model'/exp OR 'pharmacokinetic*':ti,ab,kw OR 'nonlinear mixed effect*':ti,ab,kw OR 'nonmem':ti,ab,kw OR 'WinNonlin':ti,ab,kw OR 'non-compartmental analys*':ti,ab,kw OR 'noncompartmental analys*':ti,ab,kw OR 'pharmacometric*':ti,ab,kw OR 'ADAPT 5':ti,ab,kw OR 'MONOLIX':ti,ab,kw OR 'population analy*':ti,ab,kw OR 'PDx-Pop':ti,ab,kw OR 'Pharsight':ti,ab,kw OR 'PK':ti,ab,kw OR 'PopKinetics':ti,ab,kw OR 'S-ADAPT':ti,ab,kw OR 'Simcyp':ti,ab,kw OR 'nlmixed':ti,ab,kw OR 'nlme':ti,ab,kw OR 'patient-specific modeling':ti,ab,kw OR 'hepatobiliary elimination':ti,ab,kw OR 'renal elimination':ti,ab,kw OR 'area under curve':ti,ab,kw OR 'ppk':ti,ab,kw OR 'pop pk':ti,ab,kw OR 'population pharmacokinetic*':ti,ab,kw OR 'winbugs':ti,ab,kw OR 'non compartmental analysis':ti,ab,kw
4	1 AND 2 AND 3
**Cochrane Library**	
1	(MeSH descriptor: [Voriconazole] explode all trees) OR voriconazol*:ti,ab,kw OR vorikonazol*:ti,ab,kw OR vfend*:ti,ab,kw
2	(MeSH descriptor: [Pharmacokinetics] explode all trees) OR (MeSH descriptor: [Area Under Curve] explode all trees) OR (MeSH descriptor: [Hepatobiliary Elimination] explode all trees) OR (MeSH descriptor: [Renal Elimination] explode all trees) OR (MeSH descriptor: [Tissue Distribution] explode all trees) OR pharmacokinetic*:ti,ab,kw OR (MeSH descriptor: [Models, Biological] explode all trees) OR (MeSH descriptor: [Patient-Specific Modeling] explode all trees) OR (nonlinear NEAR/1 mixed NEAR/1 effect*):ti,ab,kw OR nonmem:ti,ab,kw OR WinNonlin:ti,ab,kw OR (non-compartmental NEAR/1 analys*):ti,ab,kw OR (noncompartmental NEAR/1 analys*):ti,ab,kw OR pharmacometric*:ti,ab,kw OR (ADAPT NEAR/1 5):ti,ab,kw OR MONOLIX:ti,ab,kw OR (population NEAR/1 analy*):ti,ab,kw OR PK:ti,ab,kw OR S-ADAPT:ti,ab,kw OR Simcyp:ti,ab,kw OR nlmixed:ti,ab,kw OR nlme:ti,ab,kw
3	(pediatric* OR paediatric* OR child* OR stepchild* OR newborn* OR congenital* OR infan* OR baby OR babies OR neonat* OR pre-term OR preterm* OR pre-matur* OR prematur* OR (birth NEAR/1 weight*) OR birthweight* OR (full NEAR/1 term) OR full-term OR post-natal* OR postnatal* OR postterm OR post-term OR NICU OR preschool* OR pre-school* OR junior* OR toddler* OR kid* OR boy* OR girl* OR pubescen* OR juvenile* OR teen* OR preteen* OR pre-teen* OR youth* OR youngster* OR adolesc* OR preschool* OR school* OR kindergarten* OR pre-pubesc* OR prepubesc* OR puberty OR minors OR p?ediatric* OR PICU):ti,ab,kw OR (MeSH descriptor: [Adolescent] explode all trees) OR (MeSH descriptor: [Child] explode all trees) OR (MeSH descriptor: [Child, Preschool] explode all trees) OR (MeSH descriptor: [Infant] explode all trees) OR (MeSH descriptor: [Child, Hospitalized] explode all trees)
4	1 AND 2 AND 3
**Web of Science**	
1	voriconazole OR voriconazol* OR vorikonazol* OR vfend*
2	child* OR pediatric* OR paediatric* OR infant* OR newborn* OR neonat* OR toddler* OR preschool* OR "pre-school" OR schoolchild* OR "school child*" OR "school age*" OR schoolage OR adolescent* OR teen* OR youth* OR juvenile OR minors OR "under age" OR underage OR boy OR boys OR girl OR girls OR puberty OR pubescent* OR "young adult*" OR "young people" OR "pediatric patient*" OR "paediatric patient*" OR "pediatric population" OR "paediatric population" OR NICU OR PICU OR stepchild* OR congenital* OR baby OR babies OR pre-term OR preterm* OR pre-matur* OR prematur* OR birthweight* OR "birth weight"

	OR "birth-weight" OR "full term" OR "full-term" OR post-natal* OR postnatal* OR postterm OR "post-term" OR kindergarten* OR pre-pubesc* OR prepubesc*
3	pharmacokinetic* OR "area under curve" OR "area under the curve" OR "hepatobiliary elimination" OR "renal elimination" OR "tissue distribution" OR "biological model*" OR "patient-specific modeling" OR nonlinear NEAR/1 mixed NEAR/1 effect* OR nonmem OR WinNonlin OR non-compartmental NEAR/1 analys* OR noncompartmental NEAR/1 analys* OR pharmacometric* OR "ADAPT 5" OR MONOLIX OR population NEAR/1 analy* OR "PDx-Pop" OR Pharsight OR PK OR PopKinetics OR "S-ADAPT" OR Simcyp OR nlmixed OR nlme OR PPK OR "PPK model*" OR popPK OR "popPK model*" OR "population pharmacokinetic*" OR "nonlinear mixed effect*"
4	1 AND 2 AND 3
**Supplementary Literature**	
1	**Therapeutic Drug Monitoring** [search terms: voriconazole and ("population pharmacokinetic*" OR "population PK" OR "pharmacokinetic model*") and (pediatric OR paediatric OR child OR children OR infant OR neonate OR adolescent OR NICU OR PICU)
2	Conference proceedings in **American College of Clinical Pharmacy Conference** [search terms: ("J Am Coll Clin Pharm"[ta]) AND (voriconazole AND pharmacokinetics) AND ("congress"[pt] OR "meeting abstract"[pt] OR "conference"[pt])]

Table S2. Reporting completeness assessment based on the ClinPK checklist

Authors and Items	Shen 2025[1]	Wu 2025[2]	Shoji 2024[3]	Hu 2023[4]	Takahashi 2022[5]	Wu 2022[6]	Takahashi 2021[7]	Wang 2021[8]	Knight-Perry 2020[9]	Carlesse 2019[10]	Gastine 2018 [11]	Muto 2015[12]	*Mixed* Neely 2015[13]	Hicks 2014[14]	*Mixed* Friberg 2012[15]	Neely 2010[16]	Karlsson 2009 [17]	Walsh 2004 [18]	**Compliance**	
																			Count	%
**Title/abstract**																				
1.drug and patient	+	+	+	+	+	+	+	+	+	+	+	+	+	+	+	+	+	+	18/18	100
2.name of the drug	+	+	+	+	+	+	+	+	+	+	+	+	+	+	+	+	+	+	18/18	100
3.administration route	-	-	-	+	+	-	-	+	-	-	-	-	-	-	-	-	+	+	5/18	27.8
4.population studied	+	+	+	+	+	+	+	+	+	+	+	+	+	+	+	+	+	+	18/18	100
5.primary objective	+	+	+	+	+	+	+	+	+	+	+	+	+	+	+	+	+	+	18/18	100
6.major clinPK findings	+	+	+	+	+	+	+	+	+	+	+	+	+	+	+	+	+	+	18/18	100
**Background**																				
7.pharmacokinetic data	+	+	+	+	+	+	+	+	+	+	+	+	+	+	+	+	+	+	18/18	100
8.study rationale	+	+	+	+	+	+	+	+	+	+	+	+	+	+	+	+	+	+	18/18	100
9.specific objectives	+	+	+	+	+	+	+	+	+	+	+	+	+	+	+	+	+	+	18/18	100
**Methods**																				
10.eligibility criteria	+	+	+	+	+	+	+	+	+	+	+	+	+	+	+	+	+	+	18/18	100
11.co-administration	+	+	+	+	+	+	+	+	+	+	-	+	-	+	-	+	+	-	14/18	77.8
12.dosage	+	+	+	+	+	-	+	+	+	-	+	+	+	+	+	+	+	+	16/18	88.9
13.route	+	+	+	+	+	+	+	+	+	+	+	+	+	+	+	+	+	+	18/18	100
14.frequency	+	+	+	+	+	+	+	+	+	-	+	+	-	+	+	+	+	+	16/18	88.9
15.infusion duration	?	+	-	-	+	-	+	-	+	-	+	+	+	-	+	-	+	+	10/17	58.8
16.sample time	+	+	+	+	+	+	+	+	+	+	+	+	+	+	+	+	+	+	18/18	100
17.sample storage	-	-	-	-	-	-	+	-	+	-	+	-	+	-	-	-	-	-	4/18	22.2

18.bioanalytical assay	+	+	+	+	-	+	-	+	+	+	+	-	+	+	-	+	+	+	14/18	77.8
19.modeling methods	+	+	+	+	+	+	+	+	-	-	+	+	-	-	+	-	+	+	13/18	72.2
20.modeling software	+	+	+	+	+	+	+	+	+	+	+	+	+	+	+	+	+	+	18/18	100
21.covariates model	+	+	+	+	+	+	+	+	+	+	+	+	+	+	+	+	+	+	18/18	100
22.formulas for calculated variables	+	+	-	-	-	-	-	-	-	+	+	-	+	-	-	-	-	-	13/18	72.2
23.specific body weight	+	+	+	-	+	+	+	-	+	+	+	+	+	-	-	-	-	-	11/18	61.1
24.statistical methods and software	+	+	-	+	+	+	+	-	+	+	+	+	+	-	+	+	-	+	14/18	77.8
**Results**																				
25.study withdrawals or subjects lost	?	-	?	?	-	?	-	-	?	?	-	-	+	?	-	?	-	+	2/10	20
26.quantification of missing or excluded data	-	-	+	-	+	-	+	+	?	-	-	-	+	-	+	+	+	-	8/17	47.1
27.pharmacokinetic variability	+	+	+	+	+	+	+	+	+	+	+	+	+	+	+	+	+	+	18/18	100
28.results reported with precision	+	+	+	+	+	+	+	+	+	+	+	+	+	+	+	+	+	+	18/18	100
**Discussion/conclusion**																				
29.study limitations	+	+	+	+	+	+	+	+	+	+	+	+	+	+	+	+	+	+	18/18	100
30.study findings	+	+	+	+	+	+	+	+	+	+	+	+	+	+	+	+	+	+	18/18	100
**Other information**																				
31.funding sources	+	+	+	+	+	-	+	+	+	+	-	+	+	+	+	+	+	-	15/18	83.3
32.conflicts of interest	+	+	+	+	+	+	+	+	+	+	-	+	+	+	+	+	+	-	16/18	88.9
**Compliance (count, %)**	27/30	28/32	26/31	26/31	28/32	24/31	28/32	26/32	27/30	24/31	26/32	26/32	28/32	23/31	25/32	25/31	27/32	24/31	/	/
	90	87.5	83.9	83.9	87.5	77.4	87.5	81.3	90	77.4	81.3	81.3	87.5	74.2	78.1	80.6	84.4	77.4		

Abbreviation: ClinPK, clinical pharmacokinetic.

+ : Meet report requirements (record 1 score); - : Not reported (record zero score); ? : Not applicable

Table S3. Characteristics of individual studies.

Author and Year of Study	Sample size	Study population	Age, mean (SD) or as described	Male/female, n or %	Weight (kg), mean (SD) or as described	Observed dose/regimen	Sampling design	Routes	Total samples	Assay	CYP2C19 phenotype
Shen 2025[1]	76	Immunocompromised children under 2 years of age	median: 11 months, range: 7.38-17.00 months	58/18	median: 8.05, range: 6.95-9	4-10 mg/kg q12h; individualized by treating clinicians.	Trough only	PO	110	HPLC	NR
Wu 2025[2]	57	Children undergoing Allo-HSCT (2-18 years of age)	median: 9 years, range: 2.58-15 years	44/13	median: 20.5, IQR: 11.6-42	Clinician- or label-directed; doses varied; exact cohort doses not reported.	Trough and peak	IV/PO	237	HPLC	NM: IM: PM=27: 25: 5
Shoji 2024[3]	60	Pediatric patients with malignancy or inborn errors of immunity	median: 5.3 years, IQR: 1.4-10 years	32/28	median: 16.7, IQR: 10.4–25.1	IV: median 8.31 mg/kg/dose; PO: median 7.63 mg/kg/dose; q12h.	Trough only	IV/PO	526	HPLC and LC-MS/MS	RM: NM: IM: PM=1: 21: 27: 11
Hu 2023[4]	91	Children ≤ 14 years of age with malignant hematological diseases	median: 10 years, range: 2-14 years	58 /33	median: 31, range: 9.5-85	IV: 9 mg/kg loading dose, then 8 mg/kg q12h; PO: 9 mg/kg q12h; TDM-adjusted.	Trough only	IV/PO	210	HPLC	NM: IM: PM=37: 43: 11
Takahashi 2022[5]	59	Pediatric patients undergoing HCT (age < 21 years)	median: 11 years, range: 0.6-20 years	37 /22	median: 33, range: 5-133	IV: 6-12 mg/kg q12h; infusion rate 3 mg/kg/h.	Dense sampling (>5 samples)	IV	NR	LC-MS/MS	UM: RM: NM: IM: PM=1: 12: 31: 14: 1
Wu 2022[6]	67	Patients aged < 18 years with haematological malignancies	median: 7.83 years, range: 1.08-17.92 years	36 /31	median: 23.7, IQR: 15.95-43.5	Initial dose followed label or clinician practice; doses varied.	Trough only	IV/PO	146	EMIT	NM: IM: PM=31: 27: 9
Takahashi 2021[7]	58	Patients aged < 21 years undergoing hematopoietic cell transplantation (HCT)	median: 11 years, range: 0.6-20 years	36/22	median: 34, range: 5-133	IV q12h; infusion rate 3 mg/kg/h; trial doses varied by weight and phenotype.	Dense sampling (>5 samples)	IV	NR	LC-MS/MS	(UM+RM): NM: (IM+PM) = 13: 30: 15
Wang 2021[8]	99	Children with a critical illness	median: 5.25 years, range: 0.44-13.58 years	60/39	median: 18, range: 7-41	Loading dose: median 6 mg/kg (range 3.6-9.8); maintenance dose: median 4 mg/kg (range 3.6-9.0).	Random concentrations	IV	195	HPLC	UM: NM: IM: PM=1: 34: 45: 14
Knight-Perry 2020[9]	59	Patients aged ≤ 21 years undergoing hematopoietic cell transplantation (HCT)	median: 10 years, range: 1-21 years	NR	NR	Phase I IV dose-escalation cohorts; infusion rate 3 mg/kg/h.	Dense sampling (>5 samples)	IV	1288 for voriconazole or n-oxide concentrations	LC-MS/MS	NR
Carlesse 2019[10]	55	Pediatric patients aged < 18 years with cancer.	median: 11.2 years, IQR: 8.1-14.5 years	30/25	NR	Clinician-directed IV/PO; doses varied; exact regimen not reported.	Unspecified	IV/PO	158	LC-MS/MS	NR
Gastine 2018[11]	23	Patients aged ≤ 21 years	≤ 12 years: median: 8 years, range: 0.5-12 years;<br>> 12 years: median: 14 years, range: 13-21 years	≤ 12 years: 6/6;<br>> 12 years: 9/2	≤ 12 years: median: 27, range: 7-44;<br>> 12 years: median: 56, range: 39-85	IV/PO regimens varied across contributing source cohorts.	Trough and peak	IV/PO	187	HPLC	NR
Muto 2015[12]	21	Immunocompromised children aged 3 to 14 years	median: 10 years, range: 3-14 years	9/12	median: 31.5, range: 11.5-55.2	IV-to-oral regimens in the parent study; weight-based doses varied.	Dense sampling (>5 samples)	IV/PO	276	NR	NM: IM: PM=9: 10: 2

Author and Year of Study	Sample size	Study population	Age, mean (SD) or as described	Male/female, n or %	Weight (kg), mean (SD) or as described	Observed dose/regimen	Sampling design	Routes	Total samples	Assay	CYP2C19 phenotype
Mixed Neely 2015[13]	174: Development: 141 (85 children, 56 adults); validation: 33 children	Children and adults receiving voriconazole	Children: 5.6 years (SD: 3.0), range: 2-11 years; Adults: 31.8 years (SD: 8.4), range: 19-55 years	NR	Children: 22.7 (SD: 10.5), range: 10.8–54.9; Adults: 75.8 (SD: 9.8), range: 49–97	Clinical IV/PO regimens varied across cohorts.	Dense sampling (>5 samples)	IV/PO	NR; mean observations per subject: children 20.3, adults 36.5	LC-MS/MS	NR
Hicks 2014[14]	33	Children aged 1–19 years with cancer	median: 9 years, range: 1-19 years	19/14	≤ 12 years: median: 19.4, range: 8.6–57.8; > 12 years: median: 71.6, range: 40–110	2.6-41.2 mg/kg/day (<12 y); 3.6-16.1 mg/kg/day (>=12 y).	Trough only	IV/PO	142	HPLC and LC-MS/MS	UM: NM: IM: PM=12: 11: 9: 1
Mixed Friberg 2012[15]	173 (112 children, 26 adolescents, 35 adults)	Immunocompromised children aged 2-12 years and adolescents aged 12-17 years, and healthy adults	Children: median: 5 years, range: 2-11 years; Adolescents: median: 13 years, range: 12-16 years; Adults: median: 34 years, range: 22-55 years	Children: 60/52; Adolescents: 17/9; Adults: 24/11	Children: median: 20.1, range: 10.8–54.9; Adolescents: median: 57.1, range: 30.4-92.2; Adults: median: 76, range: 49-97	Children: IV 3-8 mg/kg q12h; oral 4-6 mg/kg q12h across cohorts.	Dense sampling (>5 samples)	IV/PO	3336 (2022 for children, 554 for adolescents, 760 for adults)	NR	Children: UM: NM: IM: PM =2: 70: 38: 2; Adolescents: UM: NM: IM: PM =2: 8: 14: 2; Adults: NM: IM: PM=20: 14: 1
Neely 2010[16]	40	Patients had voriconazole serum concentrations measured during hospitalization for a 1-year period	range: 8 months-20.5 years	65%/35%	median: 33.3, range: 6.5-102.2	Individualized IV/PO doses varied by patient.	Trough only	IV/PO	108	HPLC	NR
Karlsson 2009[17]	82	Pediatric patients of 2 to <12 years of age	range: 2-12 years	47/35	22.8, range: 10.8-54.9	IV 3-8 mg/kg q12h; oral suspension 4 or 6 mg/kg q12h across cohorts.	Dense sampling (>5 samples)	IV/PO	1274	HPLC	NM: IM: PM=58: 21: 3
Walsh 2004[18]	35	Immunocompromised pediatric patients (2 to 11 years)	6.2 years, range: 2-11 years	NR	23.4, range: 12-54	IV 3 or 4 mg/kg in single- and multiple-dose regimens; infusion rate 3 mg/kg/h.	Trough and peak	IV	355	LC-MS/MS	NM: IM: PM=22: 11: 2

Notes: Values describe characteristics, treatment, and sampling in the source datasets used for PopPK model development. Observed doses refer to doses administered in the source studies and should not be interpreted as model-derived dosing recommendations. Model-derived dosing proposals and exposure targets are summarized separately in Table S5. NR indicates not reported or unavailable.

Abbreviations: Allo-HSCT, allogeneic hematopoietic stem cell transplantation; CPIC, Clinical Pharmacogenetics Implementation Consortium; CYP2C19, cytochrome P450 2C19; EM, extensive metabolizer; EMIT, enzyme-multiplied immunoassay technique; HCT, hematopoietic cell transplantation; HEM, heterozygous extensive metabolizer; HPLC, high-performance liquid chromatography; IM, intermediate metabolizer; IQR, interquartile range; IV, intravenous administration; LC–MS/MS, liquid chromatography–tandem mass spectrometry; NM, normal metabolizer; NR, not reported; PM, poor metabolizer; PO, oral administration; RM, rapid metabolizer; SD, standard deviation; UM, ultrarapid metabolizer. For cross-study phenotype mapping, EM corresponds to NM and HEM to IM; RM and UM were not combined. q12h, every 12 h; PopPK, population pharmacokinetics; TDM, therapeutic drug monitoring.

Table S4. Summary of population pharmacokinetic model development.

Author and Year of Study	PK software	Modelling framework	Estimation method	Covariates tested	Final structural scaling and retained covariates	Error model for IIV	Scale/Unit of measure for IIV	Error model for RUV	Model-evaluation methods
Shen 2025[1]	NONMEM	NLME	FOCE-I	Age, sex, weight, height, BSA, WBC, N%, L%, RBC, HGB, PLT, CRP, ALB, TBIL, DBIL, ALT, AST, ALP, SCr, INR, D-Dimer, eGFR, co-medications (tacrolimus, sirolimus, cyclosporine A, PPI, glucocorticoids); total daily dose, therapy duration at TDM.	Weight	Exponential	variance	NR	Internal (Bootstrap, GOF, VPC)
Wu 2025[2]	NONMEM	NLME	FOCE-I	Weight, age, gender, BSA, NEU, RBC, PLT, HGB, WBC, CRP, AST, TBIL, ALT, ALB, ALP, γ-GTP, SCr, urea, CrCl; CYP2C19 genotype, postoperative days, PHT, proton pump inhibitors (OME, lansoprazole), glucocorticoids (dexamethasone, MP), immunosuppressants (tacrolimus, mycophenolate mofetil, cyclosporine)	Weight, PHT, MP	Exponential	CV%	Proportional	Internal (Bootstrap, GOF, NPDE, pcVPC)
Shoji 2024[3]	Phoenix NLME	NLME	FOCE-I	Patient background (age, gender, height, weight, underlying disease), laboratory data (AST, ALT, γ-GTP, ALB, CRP, TBIL, SCr, leukocytopenia, thrombocytopenia, neutropenia), CYP2C19 genotype, disease type (hematological malignancy vs. others including inborn errors of immunity), concomitant CYP inhibitors and inducers, and dosage form (tablet, dry syrup, IV).	Weight, CYP2C19 phenotype, γ-GTP, CRP, and dosage form	Exponential	CV%	Proportional	Internal (GOF, pcVPC)
Hu 2023[4]	NONMEM	NLME	FOCE-I	Age, sex, weight, CYP2C19 phenotype, ALB, TBIL, ALT, AST, SCr, BUN, underlying disease: malignant hematological diseases (ALL, AML, lymphoma, thalassemia, aplastic anemia, etc.), concomitant medications: PPIs (omeprazole, pantoprazole, lansoprazole), glucocorticoids (methylprednisolone, dexamethasone, prednisone), administration route (oral, intravenous, switches)	CYP2C19 phenotype	Exponential	CV%	Proportional	Internal (GOF, bootstrap)
Takahashi 2022[5]	NONMEM	NLME	FOCE-I	Weight, CYP2C19 phenotype, sex, use of proton pump inhibitor (CYP2C19 inhibitor), use of corticosteroid, age, ALB, TBIL, ALT, AST, IL-6, CRP, cyclosporine whole blood C_{min}, eGFR	Weight, CYP2C19 phenotype, CRP and ALB	Exponential	CV%	Proportional and IIV on RUV	Internal (GOF, pcVPC)
Wu 2022[6]	NONMEM	NLME	FOCE-I	Sex, age, weight, BSA, WBC, NEU, HGB, PLT, TBIL, AST, ALT, γ-GTP, ALB, ALP, SCr, cystatin C, CrCl, proton pump inhibitors (PPIs: esomeprazole, pantoprazole, lansoprazole, rabeprazole), glucocorticoids (dexamethasone, methylprednisolone), CYP2C19 genotype	Weight, CYP2C19 phenotype, and ALB	Exponential	CV%	Proportional	Internal (GOF, NPDE, bootstrap)
Takahashi 2021[7]	NONMEM	NLME	FOCE-I	Weight, age, sex, BMI, genetic variants (CYP2C19, CYP3A4*22, CYP3A5, ABCB1, ABCC2, ABCG2, CYP2C9, UGT1A4, FMO), AST, ALT, TBIL, CRP, IL-6, ALB, and drug-drug interactions (pantoprazole, corticosteroids, cyclosporine)	Weight and CYP2C19 phenotype	Exponential	CV%	Proportional	Internal (GOF, pcVPC, SIR, subgroup assessment for age <2 years)
Wang 2021[8]	Phoenix NLME	NLME	FOCE-ELS	Age, sex, height, body weight, BSA, ALT, AST, TBIL, ALB, BUN, SCr, uric acid, eGFR, CRP, IL-6, CYP2C19 genotype, ABCB1 genotype, concomitant drugs (omeprazole, glucocorticoids)	Weight, CYP2C19 phenotype and omeprazole	Exponential	CV%	Proportional	Internal (GOF, NPDE, VPC, bootstrap)

Author and Year of Study	PK software	Modelling framework	Estimation method	Covariates tested	Final structural scaling and retained covariates	Error model for IIV	Scale/Unit of measure for IIV	Error model for RUV	Model-evaluation methods
Knight-Perry 2020[9]	NONMEM	NLME	NR	Weight (allometric scaling) and age (maturation factor on first-order clearance)	Weight and age	Exponential	CV%	Proportional	Internal (GOF)
Carlesse 2019[10]	Pmetrics	NPAG	NA	Ethnic group, age, sex, weight, hepatic dysfunction	Weight	NA	NA	NA	Internal (Observed vs. Predicted concentration plots, AIC/BIC)
Gastine 2018[11]	NONMEM	NLME	FOCE-I	Age, sex, CRP, bilirubin, SCr, AST, ALT, γ-GT, ALP, weight, BSA	Weight	Exponential and logit-scale additive	CV% and variance	Proportional and additive	Internal (GOF, pcVPC)
Muto 2015[12]	NONMEM	NLME	FOCE	CYP2C19 genotype, age, sex, liver function parameters, weight, BMI	Weight and CYP2C19 phenotype	Exponential and logit-scale additive	CV% and SD	Proportional	Internal (GOF, pcVPC)
Mixed Neely 2015[13]	Pmetrics	NPAG	NA	Weight and age	Weight	NA	NA	Proportional and additive	Internal (AIC, GOF, NPDE, bias/imprecision) / External
Hicks 2014[14]	Monolix	NLME	NR	Age, ancestry (African, Asian, European, Hispanic), sex, CYP2C19 genotype, concomitant antifungals (fluconazole, posaconazole), proton pump inhibitors (omeprazole, pantoprazole), steroids (dexamethasone, hydrocortisone, methylprednisolone).	Weight, age and CYP2C19 phenotype	Exponential	CV%	NR	Internal (predictive performance via dose escalation)
Mixed Friberg 2012[15]	NONMEM	NLME	FOCE	Age, weight, CYP2C19 genotype, formulation type (powder of oral suspension or tablet), study population and study effects	Weight and age	Exponential and logit-scale additive	CV% and SD	Proportional	Internal (bootstrap, pcVPC, GOF, AUC-based predictive checks)
Neely 2010[16]	MM-USCPACK	NPAG	NA	Weight, age, sex, SCr, ALT, ALP, race, ethnicity	Weight and age	NA	NA	NA	Internal (GOF)
Karlsson 2009[17]	NONMEM	NLME	FOCE-I	Age, sex, weight, height, race, CYP2C19 genotype, underlying disease (leukemia, bone marrow transplant, aplastic anemia, lymphoma, or other), presence of mucositis, SCr, AST, ALT, ALP, γ-GTP, ALB, TBIL, TP, co-administration CYP2C19/CYP2C9/CYP3A4 inhibitors and CYP inducers	Weight, CYP2C19 phenotype, ALT	Exponential	CV%	Proportional	Internal (GOF, 10-case deletion diagnostics, comparison of predicted vs. non-compartmental AUC)
Walsh 2004[18]	NONMEM	NLME	FOCE-I	Weight, CYP2C19 genotype, ALT and ALP	Weight, CYP2C19 phenotype, ALT and ALP	Exponential	CV%	NR	Internal (cross validation)

Notes: NR indicates information not reported in the original publication; NA indicates not applicable. Candidate covariates listed as tested are distinct from effects retained in the final model; absence from the final model is not interpreted as evidence of no association.

Abbreviations: ABCB1, ATP-binding cassette subfamily B member 1; ABCC2, ATP-binding cassette subfamily C member 2; ABCG2, ATP-binding cassette subfamily G member 2; AIC, Akaike information criterion; ALB, albumin; ALL, acute lymphoblastic leukaemia; ALP, alkaline phosphatase; ALT, alanine aminotransferase; AML, acute myeloid leukaemia; AST, aspartate aminotransferase; AUC, area under the concentration–time curve; BIC, Bayesian information criterion; BMI, body mass index; BSA, body surface area; BUN, blood urea nitrogen; Cmin, minimum concentration; CrCl, creatinine clearance; CRP, C-reactive protein; CV%, coefficient of variation expressed as a percentage; CYP, cytochrome P450; CYP2C19, cytochrome P450 2C19; CYP2C9, cytochrome P450 2C9; CYP3A4, cytochrome P450 3A4; CYP3A5, cytochrome P450 3A5; DBIL, direct bilirubin; eGFR, estimated glomerular filtration rate; FMO, flavin-containing monooxygenase; FOCE, first-order conditional estimation; FOCE-ELS, first-order conditional estimation with extended least squares; FOCE-I, first-order conditional estimation with interaction; γ-GT, γ-glutamyl

transferase; γ-GTP, γ-glutamyl transpeptidase; GOF, goodness-of-fit; HGB, haemoglobin; IIV, interindividual variability; IL-6, interleukin-6; IM, intermediate metabolizer; INR, international normalized ratio; IV, intravenous administration; L%, lymphocyte percentage; MM-USCPACK, multiple-model dosage design using USC*PACK; MP, methylprednisolone; N%, neutrophil percentage; NA, not applicable; NEU, neutrophil count; NLME, nonlinear mixed-effects modelling; NM, normal metabolizer; NONMEM, nonlinear mixed-effects modelling software; NPAG, nonparametric adaptive grid; NPDE, normalized prediction distribution errors; NR, not reported; OME, omeprazole; pcVPC, prediction-corrected visual predictive check; PHT, phenytoin; PK, pharmacokinetic; PLT, platelet count; PM, poor metabolizer; PPI, proton pump inhibitor; RBC, red blood cell count; RM, rapid metabolizer; RUV, residual unexplained variability; SCr, serum creatinine; SD, standard deviation; SIR, sampling importance resampling; TBIL, total bilirubin; TDM, therapeutic drug monitoring; TP, total protein; UGT1A4, UDP-glucuronosyltransferase 1A4; UM, ultrarapid metabolizer; VPC, visual predictive check; WBC, white blood cell count.

Table S5. Summary of population pharmacokinetic model applications and dosing recommendations.

Author and Year of Study	Dosing recommendations	C_{trough}
Shen 2025[1]	Establish a machine learning (ML) model for individualized initial dose recommendation	1–5.5 mg/L
Wu 2025[2]	Co-administration with PHT (with or without MP): A dose approximately threefold higher than the conventional regimen was required during co-administration with PHT:<br>10–19 kg: 29 mg/kg loading → 27.5 mg/kg q12h;<br>20–29 kg: 27 mg/kg loading → 25 mg/kg q12h;<br>30–40 kg: 25.5 mg/kg loading → 23 mg/kg q12h.<br>Co-administration with MP only: 12.5–50% higher than no-drug group:<br>10–19 kg: 13.5 mg/kg loading → 12.0 mg/kg q12h;<br>20–29 kg: 12.5 mg/kg loading → 10.5 mg/kg q12h;<br>30–40 kg: 12 mg/kg loading → 9 mg/kg q12h.<br>No PHT or MP: Maintenance reduced to ~5 mg/kg (from 8 mg/kg):<br>10–19 kg: 9.5 mg/kg loading → 5.5 mg/kg q12h;<br>20–29 kg: 9 mg/kg loading → 5 mg/kg q12h;<br>30–40 kg: 9 mg/kg loading → 4.5 mg/kg q12h.	0.5–5 mg/L
Shoji 2024[3]	20 kg patients:<br>IV: NM/RM 11 mg/kg q12h; IM 7 mg/kg q12h; PM 3 mg/kg q12h. In the presence of γ-GTP ≥ Grade 2, the recommended dose decreases (e.g., PM to 2 mg/kg, IM to 6 mg/kg); when CRP ≥ 2.0 mg/dL or both factors are present, the dose requirements for NM/RM are similar or slightly higher (up to 12 mg/kg).<br>Oral dry syrup: NM/RM 9 mg/kg q12h; IM 6 mg/kg q12h; PM 3 mg/kg q12h.<br>Oral tablet: NM/RM 15 mg/kg q12h; IM 7 mg/kg q12h; PM 3 mg/kg q12h.<br>50 kg patients:<br>IV: NM/RM 7 mg/kg q12h; IM 5 mg/kg q12h; PM 2 mg/kg q12h. With γ-GTP ≥ Grade 2, doses are generally lower (e.g., PM 1 mg/kg, IM 3 mg/kg); with CRP ≥ 2.0 mg/dL or both factors, NM/RM doses remain at 7–8 mg/kg.<br>The maximum oral dose was capped at 300 mg.<br>Oral dry syrup / tablet: NM/RM 5–6 mg/kg q12h; IM 4 mg/kg q12h; PM 2 mg/kg q12h.	≥ 1 mg/L
Hu 2023[4]	NMs: oral 9 mg/kg (PTA 44.9%) or IV 8 mg/kg (PTA 60.9%) twice daily.<br>IMs: oral 9 mg/kg (PTA 65.7%) or IV 5 mg/kg (PTA 66.1%) twice daily.<br>PMs: oral 6 mg/kg (PTA 64.3%) or IV 5 mg/kg (PTA 56.5%) twice daily.	1.0–5.5 mg/L
Takahashi 2022[5]	NA	NA
Wu 2022[6]	Order of the recommended doses:<br>EM > IM > PM. Children with lower WT should receive a higher dose, while those with lower ALB levels should receive a lower dose.	0.5–5.0 mg/L
Takahashi 2021[7]	Every 12 hours, infusion rate 3 mg/kg/hour, stratified by body weight and CYP2C19 phenotype, without a loading dose:<br>For CYP2C19 poor/intermediate metabolizers: Weight < 15 kg: 8 mg/kg every 12 h; Weight 15–30 kg: 8 mg/kg every 12 h; Weight > 30 kg: 6 mg/kg every 12 h<br>For CYP2C19 normal metabolizers: Weight < 15 kg: 16 mg/kg every 12 h; Weight 15–30 kg: 12 mg/kg every 12 h; Weight > 30 kg: 10 mg/kg every 12 h<br>For CYP2C19 rapid/ultrarapid metabolizers: Weight < 15 kg: 20 mg/kg every 12 h; Weight 15–30 kg: 18 mg/kg every 12 h; Weight > 30 kg: 14 mg/kg every 12 h	1.5–5.0 mg/L
Wang 2021[8]	The order of recommended doses by CYP2C19 phenotype is: EM (extensive metabolizer) > IM (intermediate metabolizer) > PM (poor metabolizer). Children with lower body weight (WT) require higher mg/kg doses.<br>For loading doses, in children weighing ≤18 kg with EM/IM phenotypes, administering every 8 hours (q8h) on the first day provides a higher probability of target attainment (PTA) than a q12h loading regimen.<br>Maintenance dose: Administered every 12 hours (q12h) after the first day.<br>PTA for the recommended regimens: Ranges from 72.2% to 99%	1.0–5.5 mg/L
Knight-Perry 2020[9]	< 2 years: loading dose of 12 mg/kg q12h for 2 doses, followed by maintenance dosing of 10 mg/kg q12h.<br>2–11 years: starting dose of 10 mg/kg q12h (no loading dose specified).	1.5–5.0 mg/L

Author and Year of Study	Dosing recommendations	C_{trough}
	12–21 years: no MTD/MED identified; highest tested dose was 8 mg/kg q12h (achieved therapeutic trough in only 50% of patients).	
Carlesse 2019[10]	NA	1.0–6.0 mg/L
Gastine 2018[11]	A simulation-derived loading dose of 9 mg/kg three times daily (TID) for the first 24, 48, or up to 72 hours, followed by a maintenance dose of 8 mg/kg twice daily (BID).	1.0–6.0 mg/L
Muto 2015[12]	Weight < 50 kg (age 2–<12 years, or 12–<15 years and weight <50 kg): IV maintenance: 8 mg/kg q12h (loading dose: 9 mg/kg q12h); oral maintenance: 9 mg/kg q12h (max 350 mg/dose)<br>Weight ⩾ 50 kg (age 12–<15 years and weight ⩾50 kg): IV maintenance: 4 mg/kg q12h; oral maintenance: 200 mg q12h	NA
Neely 2015[13]	NA	1.0–6.0 mg/L
Hicks 2014[14]	For patients <12 years of age: CYP2C19*17/*17: 36 mg/kg/day; CYP2C19*1/*17: 30 mg/kg/day; CYP2C19*1/*1: 20 mg/kg/day; CYP2C19*1/*2A or *1/*2B: 18 mg/kg/day<br>For patients ≥12 years of age: CYP2C19*17/*17: 28 mg/kg/day; CYP2C19*1/*17: 14 mg/kg/day; CYP2C19*1/*1: 14 mg/kg/day; CYP2C19*1/*2A or *1/*2B: 10 mg/kg/day; CYP2C19*2A/*2A: 4 mg/kg/day	1.0–6.0 mg/L
Friberg 2012[15]	Children (2 to <12 years old):<br>Intravenous loading dose: 9 mg/kg q12h to match adult 6 mg/kg loading dose. Intravenous maintenance dose: 8 mg/kg q12h to match adult 4 mg/kg q12h; also 4 mg/kg q12h in children matches adult 3 mg/kg q12h.<br>Oral maintenance dose: 9 mg/kg q12h (maximum 350 mg per dose) to match adult 200 mg oral q12h.<br>Adolescents 12–14 years old:<br>If body weight <50 kg: dose like children.<br>If body weight ⩾50 kg: dose like adults.<br>Other adolescents (15–16 years old, regardless of weight): dose like adults.<br>Adults:<br>Intravenous loading: 6 mg/kg q12h on day 1. Intravenous maintenance: 4 mg/kg q12h.<br>Oral maintenance: 200 mg q12h; also 3 mg/kg IV q12h as alternative.	NA
Neely 2010[16]	7 mg/kg IV or 200 mg PO q12h	≥ 1 mg/L
Karlsson 2009[17]	Intravenous: 7 mg/kg twice daily (BID) for children aged 2 to <12 years;<br>Oral: 200 mg BID (irrespective of body weight) for the same age group.	NA
Walsh 2004[18]	Children (2–11 years) should receive 4 mg/kg intravenously every 12 hours as maintenance dose to match adult exposure at 3 mg/kg every 12 hours.	NA

Abbreviations: ALB, albumin; BID, twice daily; CRP, C-reactive protein; Ctrough, trough concentration; CYP2C19, cytochrome P450 2C19; EM, extensive metabolizer; HEM, heterozygous extensive metabolizer; γ-GTP, γ-glutamyl transpeptidase; IM, intermediate metabolizer; IV, intravenous administration; MED, minimum effective dose; ML, machine learning; MP, methylprednisolone; MTD, maximum tolerated dose; NA, not applicable; NM, normal metabolizer; PHT, phenytoin; PM, poor metabolizer; PO, oral administration; PTA, probability of target attainment; q12h, every 12 h; q8h, every 8 h; RM, rapid metabolizer; TID, three times daily; UM, ultrarapid metabolizer; WT, body weight. For cross-study phenotype interpretation, EM corresponds to NM and HEM to IM; RM and UM remain distinct.

Table S6. Reports excluded after full-text eligibility assessment and primary reasons for exclusion.

No.	Citation	Primary reason for exclusion
**Non-PopPK analyses or individual/subject-level PK profiles (15 records)**		
1	Hikino, K.; Shoji, K.; Saito, J.; Fukunaga, K.; Liu, X.; Matsui, T.; Utano, T.; Takebayashi, A.; Tomizawa, D.; Kato, M.; et al. Analysis of factors influencing the relationship between voriconazole plasma concentrations and adverse effects in a paediatric population. *Br. J. Clin. Pharmacol.* **2025**, *91*, 2020–2027.	Secondary report of an included PopPK study.
2	Hope, W.W.; Walsh, T.J.; Goodwin, J.; Peloquin, C.A.; Howard, A.; Kurtzberg, J.; Mendizabal, A.; Confer, D.L.; Bulitta, J.; Baden, L.R.; et al. Voriconazole pharmacokinetics following HSCT: results from the BMT CTN 0101 trial. *J. Antimicrob. Chemother.* **2016**, *71*, 2234–2240.	No independently estimated PopPK model.
3	Markantonis, S.L.; Katelari, A.; Pappa, E.; Doudounakis, S. Voriconazole pharmacokinetics and photosensitivity in children with cystic fibrosis. *J. Cyst. Fibros.* **2012**, *11*, 246–252.	Conventional PK analysis only.
4	Allegra, S.; Fatiguso, G.; De Francia, S.; Favata, F.; Pirro, E.; Carcieri, C.; De Nicolò, A.; Cusato, J.; Di Perri, G.; D'Avolio, A. Therapeutic drug monitoring of voriconazole for treatment and prophylaxis of invasive fungal infection in children. *Br. J. Clin. Pharmacol.* **2018**, *84*, 197–203.	No eligible PopPK analysis.
5	Boast, A.; Curtis, N.; Cranswick, N.; Gwee, A. Voriconazole dosing and therapeutic drug monitoring in children: experience from a paediatric tertiary care centre. *J. Antimicrob. Chemother.* **2016**, *71*, 2031–2036.	No eligible PopPK analysis.
6	Chuwongwattana, S.; Jantararoungtong, T.; Prommas, S.; Medhasi, S.; Puangpetch, A.; Sukasem, C. Impact of CYP2C19, CYP3A4, ABCB1, and FMO3 genotypes on plasma voriconazole in Thai patients with invasive fungal infections. *Pharmacol. Res. Perspect.* **2020**, *8*, e00665.	No eligible PopPK analysis.
7	Driscoll, T.A.; Frangoul, H.; Nemecek, E.R.; Murphey, D.K.; Yu, L.C.; Blumer, J.; Krance, R.A.; Baruch, A.; Liu, P. Comparison of pharmacokinetics and safety of voriconazole intravenous-to-oral switch in immunocompromised adolescents and healthy adults. *Antimicrob. Agents Chemother.* **2011**, *55*, 5780–5789.	Noncompartmental PK analysis only.
8	Driscoll, T.A.; Yu, L.C.; Frangoul, H.; Krance, R.A.; Nemecek, E.; Blumer, J.; Arrieta, A.; Graham, M.L.; Bradfield, S.M.; Baruch, A.; et al. Comparison of pharmacokinetics and safety of voriconazole intravenous-to-oral switch in immunocompromised children and healthy adults. *Antimicrob. Agents Chemother.* **2011**, *55*, 5770–5779.	Noncompartmental PK analysis only.
9	Chen, J.; Wu, Y.; He, Y.; Feng, X.; Ren, Y.; Liu, S. Combined Effect of CYP2C19 Genetic Polymorphisms and C-Reactive Protein on Voriconazole Exposure and Dosing in Immunocompromised Children. *Front. Pediatr.* **2022**, *10*, 846411.	No eligible PopPK analysis.
10	Gastine, S.E.; Rauwolf, K.K.; Pieper, S.; Hempel, G.; Lehrnbecher, T.; Tragiannidis, A.; Groll, A.H. Voriconazole plasma concentrations and dosing in paediatric patients below 24 months of age. *Mycoses* **2023**, *66*, 969–976.	No eligible PopPK analysis.
11	Liu, L.; Zhou, X.; Wu, T.; Jiang, H.; Yang, S.; Zhang, Y. Dose optimisation of voriconazole with therapeutic drug monitoring in children: a single-centre experience in China. *Int. J. Antimicrob. Agents* **2017**, *49*, 483–487.	No eligible PopPK analysis.
12	Kruizinga, M.D.; Stuurman, F.E.; Driessen, G.J.A.; Cohen, A.F.; Bergmann, K.R.; van Esdonk, M.J. Theoretical Performance of Nonlinear Mixed-Effect Models Incorporating Saliva as an Alternative Sampling Matrix for Therapeutic Drug Monitoring in Pediatrics: A Simulation Study. *Ther. Drug Monit.* **2021**, *43*, 546–554.	Simulation using previously published models only.
13	Mori, M.; Fukushima, K.; Miharu, M.; Goto, H.; Yoshida, M.; Shoji, S. A retrospective analysis of voriconazole pharmacokinetics in Japanese pediatric and adolescent patients. *J. Infect. Chemother* **2013**, *19*, 174–179.	Conventional PK analysis only.
14	Mori, M.; Kobayashi, R.; Kato, K.; Maeda, N.; Fukushima, K.; Goto, H.; Inoue, M.; Muto, C.; Okayama, A.; Watanabe, K.; et al. Pharmacokinetics and safety of voriconazole intravenous-to-oral switch regimens in immunocompromised Japanese pediatric patients. *Antimicrob. Agents Chemother.* **2015**, *59*, 1004–1013.	Noncompartmental PK analysis only.
15	Takahashi, T.; Smith, A.R.; Jacobson, P.A.; Fisher, J.; Rubin, N.T.; Kirstein, M.N. Impact of Obesity on Voriconazole Pharmacokinetics among Pediatric Hematopoietic Cell Transplant Recipients. *Antimicrob. Agents Chemother.* **2020**, *64*, e00653-20.	Noncompartmental PK analysis only.
**Adult-only/no extractable pediatric-specific information (7 records)**		
16	Li, Z.W.; Peng, F.H.; Yan, M.; Liang, W.; Liu, X.L.; Wu, Y.Q.; Lin, X.B.; Tan, S.L.; Wang, F.; Xu, P.; et al. Impact of CYP2C19 Genotype and Liver Function on Voriconazole Pharmacokinetics in Renal Transplant Recipients. *Ther. Drug Monit.* **2017**, *39*, 422–428.	No extractable pediatric-specific PopPK results.
17	Lin, X.B.; Lui, K.Y.; Guo, P.H.; Liu, X.M.; Liang, T.; Hu, X.G.; Tong, L.; Wu, J.J.; Xia, Y.Z.; Chen, P.; et al. Population pharmacokinetic model-guided optimization of intravenous voriconazole dosing regimens in critically ill patients with liver dysfunction. *Pharmacotherapy* **2022**, *42*, 23–33.	No extractable pediatric-specific PopPK results.

18	Yun, J.; Bai, H.; Wang, Z.; Ma, Y.; Liu, W. Serum retinol binding protein as a novel marker for clearance and dosage optimization: pharmacokinetics study of voriconazole in a cirrhosis population. *Front. Pharmacol.* **2025**, *16*, 1543323.	No extractable pediatric-specific PopPK results.
19	Kim, H.Y.; Märtson, A.G.; Dreesen, E.; Spriet, I.; Wicha, S.G.; McLachlan, A.J.; Alffenaar, J.W. Saliva for Precision Dosing of Antifungal Drugs: Saliva Population PK Model for Voriconazole Based on a Systematic Review. *Front. Pharmacol.* **2020**, *11*, 894.	No extractable pediatric-specific PopPK results.
20	Wang, T.; Chen, S.; Sun, J.; Cai, J.; Cheng, X.; Dong, H.; Wang, X.; Xing, J.; Dong, W.; Yao, H.; et al. Identification of factors influencing the pharmacokinetics of voriconazole and the optimization of dosage regimens based on Monte Carlo simulation in patients with invasive fungal infections. *J. Antimicrob. Chemother.* **2014**, *69*, 463–470.	Adult-only population.
21	Ren, Q.X.; Li, X.G.; Mu, J.S.; Bi, J.F.; Du, C.H.; Wang, Y.H.; Zhu, H.; Lv, P.; Zhao, Q.G. Population Pharmacokinetics of Voriconazole and Optimization of Dosage Regimens Based on Monte Carlo Simulation in Patients With Liver Cirrhosis. *J. Pharm. Sci.* **2019**, *108*, 3923–3931.	No extractable pediatric-specific PopPK results.
22	Wang, T.; Yan, M.; Tang, D.; Dong, Y.; Zhu, L.; Du, Q.; Sun, D.; Xing, J.; Dong, Y. Using Child-Pugh Class to Optimize Voriconazole Dosage Regimens and Improve Safety in Patients with Liver Cirrhosis: Insights from a Population Pharmacokinetic Model-based Analysis. *Pharmacotherapy* **2021**, *41*, 172–183.	Adult-only population.
**Conference abstracts with insufficient methodological/final-model details (6 records)**		
23	González, C.; Rojas, M.J.; Villena, R.; Parra, A.; Zubieta, M.; Salgado, C.; Acuña, M.; Benadof, D. Therapeutic drug monitoring of voriconazole in children with cancer. Pharmacotherapy **2015**, *35*, E203. Meeting abstract 120.	Insufficient final PopPK model information.
24	Neely, M.; Fu, X.; Margol, A.; Rushing, T.; Liu, S.; Louie, S. Voriconazole Dosing in Children Under Two Years. Clin. Pharmacol. Drug Dev. **2016**, *5* (Suppl. 1), Poster 078.	Insufficient final PopPK model information.
25	Biltaji, E.; Korgenski, E.K.; Sherwin, C.M.T.; Constance, J.E. Voriconazole Therapeutic Target Attainment Among Pediatric Patients. Clin. Pharmacol. Drug Dev. **2017**, *6* (Suppl. 1), Poster 115.	No eligible PopPK analysis.
26	Alharbi, A.; Smith, A.; Jacobson, P.; Fisher, J.; Rubin, N.; Chan, W.; Kirstein, M. Pharmacokinetic Assessment of Voriconazole and N-oxide Metabolite in Pediatric Hematopoietic Stem Cell Transplant Recipients. Clin. Pharmacol. Drug Dev. **2018**, *7* (Suppl. 1), Poster 023.	Insufficient final PopPK model information.
27	Takahashi, T.; Smith, A.R.; Jacobson, P.A.; Fisher, J.; Rubin, N.; Kirstein, M. Prophylactic Voriconazole in Pediatric Hematopoietic Stem Cell Transplant Patients: A Pharmacokinetic Study and Therapeutic Drug Monitoring. Clin. Pharmacol. Drug Dev. **2019**, *8* (Suppl. 1), Poster 050.	Insufficient final PopPK model information.
28	Mohamud, M.; Alharbi, A.; Takahashi, T.; Smith, A.R.; Jacobson, P.A.; Fisher, J.; Rubin, N.; Kirstein, M. The Role of CYP2C19 Genetic Variants on Voriconazole Pharmacokinetics in Pediatric Hematopoietic Stem Cell Transplant Recipients. Clin. Pharmacol. Drug Dev. **2019**, *8* (Suppl. 1), Poster 110.	Insufficient final PopPK model information.
**Reviews (5 records)**		
29	Job, K.M.; Olson, J.; Stockmann, C.; Constance, J.E.; Enioutina, E.Y.; Rower, J.E.; Linakis, M.W.; Balch, A.H.; Yu, T.; Liu, X.; et al. Pharmacodynamic studies of voriconazole: informing the clinical management of invasive fungal infections. *Expert Rev. Anti-Infect. Ther.* **2016**, *14*, 731–746.	Review article.
30	Kadam, R.S.; Van Den Anker, J.N. Pediatric Clinical Pharmacology of Voriconazole: Role of Pharmacokinetic/Pharmacodynamic Modeling in Pharmacotherapy. *Clin. Pharmacokinet.* **2016**, *55*, 1031–1043.	Review article.
31	Bury, D.; Tissing, W.J.E.; Muilwijk, E.W.; Wolfs, T.F.W.; Brüggemann, R.J. Clinical Pharmacokinetics of Triazoles in Pediatric Patients. *Clin. Pharmacokinet.* **2021**, *60*, 1103–1147.	Review article.
32	Zhong, X.; Tong, X.; Ju, Y.; Du, X.; Li, Y. Interpersonal Factors in the Pharmacokinetics and Pharmacodynamics of Voriconazole: Are CYP2C19 Genotypes Enough for Us to Make a Clinical Decision? *Curr. Drug Metab.* **2018**, *19*, 1152–1158.	Review article.
33	Hon, K.L.E.; Chan, V.P.; Leung, A.K.; Leung, K.K.Y.; Hui, W.F. Invasive fungal infections in critically ill children: epidemiology, risk factors and antifungal drugs. *Drugs Context* **2024**, *13*, 2023-9-2.	Review article.
**Protocol/editorial/commentary/letter (2 records)**		
34	Johnson, T.N.; Salem, F.; Jamei, M.; Rostami-Hodjegan, A. Prediction of Voriconazole Non-linear Pharmacokinetics Using a Paediatric Physiologically Based Pharmacokinetic Modelling Approach. *Clin. Pharmacokinet.* **2015**, *54*, 567–568.	Letter or commentary.

35	Zane, N.R.; Thakker, D.R. Response to: "Prediction of Voriconazole Non-Linear Pharmacokinetics Using a Paediatric Physiologically Based Pharmacokinetic Modelling Approach". *Clin. Pharmacokinet.* **2015**, *54*, 569–572.	Letter or commentary.
**Animal study (1 record)**		
36	Elizondo-Zertuche, M.; Montoya, A.M.; Robledo-Leal, E.; Garza-Veloz, I.; Sánchez-Núñez, A.L.; Ballesteros-Elizondo, R.; González, G.M. Comparative Pathogenicity of Lomentospora prolificans (Scedosporium prolificans) Isolates from Mexican Patients. *Mycopathologia* **2017**, *182*, 681–689.	Animal study.

Notes: Where more than one criterion applies, the primary reason is shown. PopPK, population pharmacokinetics; PK, pharmacokinetics.

References

1. Shen, L.; Hu, M.; Xu, X.; Zhou, Y.; Wu, W.; Ge, X.; Wang, G.; Wang, Y.; Li, Z. Precision dosing of voriconazole in immunocompromised children under 2 years: Integrated machine learning and population pharmacokinetic modeling. *Front. Pharmacol.* **2025**, *16*, 1671652. https://doi.org/10.3389/fphar.2025.1671652.
2. Wu, Y.; Niu, L.; Ling, Y.; Zhou, S.; Huang, T.; Qi, J.; Wu, D.-N.; Cai, R.-D.; Wu, T.-Q.; Xiao, Y.; et al. Drug–drug interaction of phenytoin sodium and methylprednisolone on voriconazole: A population pharmacokinetic model in children with thalassemia undergoing allogeneic hematopoietic stem cell transplantation. *Eur. J. Clin. Pharmacol.* **2025**, *81*, 365–374. https://doi.org/10.1007/s00228-024-03795-2.
3. Shoji, K.; Hikino, K.; Saito, J.; Matsui, T.; Utano, T.; Takebayashi, A.; Tomizawa, D.; Kato, M.; Matsumoto, K.; Ishikawa, T.; et al. Pharmacogenetic implementation for CYP2C19 and pharmacokinetics of voriconazole in children with malignancy or inborn errors of immunity. *J. Infect. Chemother.* **2024**, *30*, 1280–1288. https://doi.org/10.1016/j.jiac.2024.06.009.
4. Hu, L.; Huang, S.; Huang, Q.; Huang, J.; Feng, Z.; He, G. Population pharmacokinetics of voriconazole and the role of CYP2C19 genotype on treatment optimization in pediatric patients. *PLoS ONE* **2023**, *18*, e0288794. https://doi.org/10.1371/journal.pone.0288794.
5. Wu, Y.; Lv, C.; Wu, D.; Qi, J.; Cai, R.; Zhou, S.; Li, C.; Wei, Y.; Liu, T. Dosage optimization of voriconazole in children with haematological malignancies based on population pharmacokinetics. *J. Clin. Pharm. Ther.* **2022**, *47*, 2245–2254. https://doi.org/10.1111/jcpt.13801.
6. Takahashi, T.; Jaber, M.M.; Smith, A.R.; Jacobson, P.A.; Fisher, J.; Kirstein, M.N. Predictive Value of C-Reactive Protein and Albumin for Temporal Within-Individual Pharmacokinetic Variability of Voriconazole in Pediatric Patients Undergoing Hematopoietic Cell Transplantation. *J. Clin. Pharmacol.* **2022**, *62*, 855–862. https://doi.org/10.1002/jcph.2024.
7. Wang, J.; Xu, H.; Li, R.; Wu, S.; Zou, J.; Wang, Y. Model-Oriented Dose Optimization of Voriconazole in Critically Ill Children. *Antimicrob. Agents Chemother.* **2021**, *65*, e00493-21. https://doi.org/10.1128/AAC.00493-21.
8. Takahashi, T.; Mohamud, M.A.; Smith, A.R.; Jacobson, P.A.; Jaber, M.M.; Alharbi, A.F.; Fisher, J.; Kirstein, M.N. CYP2C19 Phenotype and Body Weight-Guided Voriconazole Initial Dose in Infants and Children after Hematopoietic Cell Transplantation. *Antimicrob. Agents Chemother.* **2021**, *65*, e00623-21. https://doi.org/10.1128/AAC.00623-21.
9. Knight-Perry, J.; Jennissen, C.; Long, S.E.; Hage, S.; DeFor, T.E.; Chan, W.T.; Fisher, J.; Kirstein, M.N.; Smith, A.R. A phase I dose finding study of intravenous voriconazole in pediatric patients undergoing hematopoietic cell transplantation. *Bone Marrow Transplant.* **2020**, *55*, 955–964. https://doi.org/10.1038/s41409-019-0757-0.
10. Carlesse, F.A.D.M.C.; De Araujo, O.R.; Marques, L.M.A.; Silva, D.C.B.D.; Senerchia, A.A.; Petrilli, A.S. A pharmacokinetic model for voriconazole in a highly diversified population of children and adolescents with cancer. *Mycoses* **2019**, *62*, 399–404. https://doi.org/10.1111/myc.12899.
11. Gastine, S.; Lehrnbecher, T.; Müller, C.; Farowski, F.; Bader, P.; Ullmann-Moskovits, J.; Cornely, O.A.; Groll, A.H.; Hempel, G. Pharmacokinetic Modeling of Voriconazole To Develop an Alternative Dosing Regimen in Children. *Antimicrob. Agents Chemother.* **2018**, *62*, e01194-17. https://doi.org/10.1128/AAC.01194-17.
12. Neely, M.; Margol, A.; Fu, X.; Van Guilder, M.; Bayard, D.; Schumitzky, A.; Orbach, R.; Liu, S.; Louie, S.; Hope, W. Achieving Target Voriconazole Concentrations More Accurately in Children and Adolescents. *Antimicrob. Agents Chemother.* **2015**, *59*, 3090–3097. https://doi.org/10.1128/AAC.00032-15.
13. Muto, C.; Shoji, S.; Tomono, Y.; Liu, P. Population Pharmacokinetic Analysis of Voriconazole from a Pharmacokinetic Study with Immunocompromised Japanese Pediatric Subjects. *Antimicrob. Agents Chemother.* **2015**, *59*, 3216–3223. https://doi.org/10.1128/AAC.04993-14.

14. Hicks, J.K.; Crews, K.R.; Flynn, P.; Haidar, C.E.; Daniels, C.C.; Yang, W.; Panetta, J.C.; Pei, D.; Scott, J.R.; Molinelli, A.R.; et al. Voriconazole Plasma Concentrations in Immunocompromised Pediatric Patients Vary by *CYP2C19* Diplotypes. *Pharmacogenomics* **2014**, *15*, 1065–1078. https://doi.org/10.2217/pgs.14.53.
15. Friberg, L.E.; Ravva, P.; Karlsson, M.O.; Liu, P. Integrated Population Pharmacokinetic Analysis of Voriconazole in Children, Adolescents, and Adults. *Antimicrob. Agents Chemother.* **2012**, *56*, 3032–3042. https://doi.org/10.1128/AAC.05761-11.
16. Neely, M.; Rushing, T.; Kovacs, A.; Jelliffe, R.; Hoffman, J. Voriconazole Pharmacokinetics and Pharmacodynamics in Children. *Clin. Infect. Dis.* **2010**, *50*, 27–36. https://doi.org/10.1086/648679.
17. Karlsson, M.O.; Lutsar, I.; Milligan, P.A. Population Pharmacokinetic Analysis of Voriconazole Plasma Concentration Data from Pediatric Studies. *Antimicrob. Agents Chemother.* **2009**, *53*, 935–944. https://doi.org/10.1128/AAC.00751-08.
18. Walsh, T.J.; Karlsson, M.O.; Driscoll, T.; Arguedas, A.G.; Adamson, P.; Saez-Llorens, X.; Vora, A.J.; Arrieta, A.C.; Blumer, J.; Lutsar, I.; et al. Pharmacokinetics and Safety of Intravenous Voriconazole in Children after Single- or Multiple-Dose Administration. *Antimicrob. Agents Chemother.* **2004**, *48*, 2166–2172. https://doi.org/10.1128/AAC.48.6.2166-2172.2004.
